# Histone H4K8 lactylation promotes glioblastoma progression by inducing NUPR1-mediated autophagosome‒lysosome fusion

**DOI:** 10.7150/thno.126579

**Published:** 2026-02-04

**Authors:** Jiangli Zhao, Xuchen Liu, Yanya He, Qingyuan Sun, Zhiwei Xue, Ziyi Tang, Junzhi Liu, Jiwei Wang, Chao Li, Xinyu Wang, Ning Yang, Chen Qiu

**Affiliations:** 1 Department of Neurosurgery, Qilu Hospital, Cheeloo College of Medicine and Institute of Brain and Brain-Inspired Science, Shandong University, 250012 Jinan, China.; 2 Department of Neurosurgery, Second Affiliated Hospital, School of Medicine, Zhejiang University, 310009 Hangzhou, Zhejiang, China.; 3 Jinan Microecological Biomedicine Shandong Laboratory and Shandong Key Laboratory of Brain Function Remodeling, 250117 Jinan, China.; 4 Innovation Center for Neurological Disorders and Department of Neurology, Xuanwu Hospital, Capital Medical University, National Clinical Research Center for Geriatric Diseases, Beijing, 100053, China.; 5 Department of Radiation Oncology, Qilu Hospital, Cheeloo College of Medicine, Shandong University, Jinan, 250012, China.

**Keywords:** glioblastoma, lactylation, metabolic reprogramming, NUPR1, ZZW-115

## Abstract

**Rationale:** Glioblastoma (GBM), an aggressive malignant brain tumour associated with a dismal prognosis, is characterized by metabolic reprogramming that drives tumour progression, with the Warburg effect being a central contributor. This effect not only causes significant lactate buildup but also fuels lactylation, a novel post-translational modification implicated in the development of gliomas and various other cancers. Nevertheless, the exact molecular mechanisms by which lactylation promotes GBM progression remain largely elusive.

**Methods:** Lactylation levels in normal brain and GBM tissues were analysed using immunohistochemistry, immunofluorescence, and Western blotting. Glycolysis inhibitors and LDHA/LDHB knockdown were used to modulate histone lactylation in subsequent *in vitro* and *in vivo* experiments assessing GBM cell proliferation, invasion, and migration. CUT&Tag and RNA sequencing were used to identify H4K8la target genes, and NUPR1 expression was validated via ChIP‒qPCR and Western blotting. Autophagic flux was examined using transmission electron microscopy, EGFP-mCherry-LC3B probes, and LysoTracker staining. The therapeutic effects of NUPR1 inhibitor ZZW-115 were evaluated in both cellular and animal models.

**Results:** Histone lactylation, notably that of H4K8la, was markedly increased in GBM cells. Targeting lactate metabolism and lactylation levels attenuated GBM malignancy *in vitro* and *in vivo*. Genome-wide analysis revealed H4K8la enrichment at promoter regions, where it transcriptionally activated the autophagy regulator NUPR1. Functionally, NUPR1 enhanced protective autophagy via autophagosome‒lysosome fusion. Pharmacological inhibition of NUPR1 with ZZW-115 suppressed GBM growth by impairing autophagic flux, demonstrating therapeutic potential.

**Conclusion:** In summary, this study defines the functional and prognostic significance of histone lactylation in the progression of GBM. We identified the H4K8la-NUPR1 axis as a key regulatory pathway that mediates protective autophagy and developed targeted therapeutic strategies to disrupt this pathway. These findings provide novel insights into epigenetic regulation and targeted therapy for GBM.

## Introduction

Glioblastoma stands as the most aggressive and prevalent primary brain tumor in adults, represents one of the most treatment-refractory neurosurgical diseases, with median survival typically around 15 months even after receiving standard multimodality care 1. The current standard treatment for GBM centers on maximal resection, combined with adjuvant radiotherapy and chemotherapy. However, therapeutic outcomes are still extremely limited 2. A major barrier in glioma treatment lies in its aggressive invasiveness and intrinsic drug resistance 3, 4. Identifying molecular drivers of GBM progression and developing targeted interventions are critical to overcoming the limitations of conventional therapies. Growing evidence highlights metabolic reprogramming and epigenetic dysregulation as key mechanisms underlying GBM pathogenesis and therapy resistance 5-7.

A hallmark of metabolic reprogramming in GBM is aerobic glycolysis (the Warburg effect), which drives GBM cells to preferentially utilize glycolysis over mitochondrial oxidative phosphorylation (OXPHOS) for energy production even under oxygen-sufficient conditions 8, 9. This metabolic shift leads to copious lactate secretion and accumulation within the tumor microenvironment (TME) 10, 11. Contrary to its historical perception as a metabolically inert byproduct, lactate is now recognized as a functional signalling molecule that modulates tumor progression through immune regulation, angiogenesis, and epigenetic remodelling 12-14. Recent advances in posttranslational modifications (PTMs) have revealed that lactate functions as a substrate for histone lactylation, a novel epigenetic modification in which lactyl groups derived from lactate are covalently conjugated to lysine residues on histones, thereby directly modulating chromatin structure and transcriptional activity 15. Emerging evidence has demonstrated that lactylation plays a pivotal role in macrophage polarization, intratumoral immunoregulation, and cellular adaptive responses to metabolic stress 16-18. Notably, elevated lactate levels within the TME drive site-specific histone lactylation, which in turn upregulates oncogenic gene expression while concurrently suppressing antitumor immunity. Multiple research teams have confirmed that lactate-derived lactylation acts on histones such as H3 and H4, participating in the regulation of various physiological functions and playing a significant role in the malignant progression of multiple tumors 19, 20. Histone lactylation exerts regulatory functions by targeting key enzymes and metabolic pathways, thereby influencing critical biological processes, including apoptosis, autophagy, and pyroptosis, which collectively contribute to the modulation of disease progression 21, 22. Existing studies suggest that histone lactylation is among the factors that promote the progression of GBM 23, 24. However, the precise mechanisms by which tumor metabolic reprogramming promotes phenotypic regulation, particularly key histone lactylation sites, and the underlying molecular mechanisms in GBM remain incompletely understood.

Macroautophagy/autophagy, a highly conserved lysosomal degradation pathway, involves the encapsulation of damaged organelles and abnormal proteins within double-membrane autophagosomes; subsequent autophagosome-lysosome fusion degrades these components, maintaining cellular homeostasis 25. In the tumor microenvironment, autophagy has dual functions: in the early stages of carcinogenesis, it suppresses malignant transformation by clearing damaged components; conversely, in advanced stages, it promotes tumor survival by mediating immune evasion and therapy resistance 26, 27. Recent research has prioritized understanding the regulatory role of autophagy in tumor progression. Numerous autophagy-related (ATG) processes have been implicated in diverse tumor pathophysiologies 28, 29. Previous research by our group has demonstrated that BCL2L13, a key autophagy-related gene, promotes the progression of GBM by mediating mitochondrial fission to induce mitophagy 30. Existing studies suggest that the activation of lactylation in tumor cells can influence the process of autophagy, thereby regulating tumor progression 31, 32. The regulatory function of histone lactylation in autophagy during GBM progression, however, remains largely unclear.

NUPR1 (nuclear protein 1/p8/Com1) is a stress-inducible nuclear transcription regulator involved in diverse physiological processes 33. NUPR1 is overexpressed in various malignant human tumors (lung cancer, liver cancer, bladder cancer, pancreatic ductal adenocarcinoma, etc.) and promotes tumor progression through different mechanisms 34, 35. Emerging evidence has established NUPR1 as an autophagy modulator that enhances tumor survival by regulating autophagic flux 36. Our preliminary findings suggest that specific blockade of H4K8 lactylation leads to a marked downregulation of NUPR1 expression. Nevertheless, the precise mechanistic involvement of NUPR1 in glioma pathogenesis and the potential existence of analogous autophagy regulatory pathways remain to be fully elucidated.

This study elucidates the role of H4K8la in GBM progression through NUPR1-mediated protective autophagy. We demonstrate that Warburg effect-driven lactate accumulation promotes the expression of H4K8la, which activates the oncogene NUPR1 to increase autophagic flux by facilitating autophagosome‒lysosome fusion, thereby fuelling GBM growth and invasion. Importantly, the NUPR1 inhibitor ZZW-115 effectively blocked this pathway, resulting in significant antitumor effects in preclinical models. These findings reveal a lactylation-NUPR1-autophagy axis in GBM and identify a promising therapeutic target.

## Materials & Methods

### Clinical tissue samples

Different WHO grade glioma samples were obtained from intraoperative resections of 20 patients undergoing surgery at Qilu Hospital of Shandong University (2022-2025). 10 Normal brain tissues were collected from traumatic brain injury patients receiving decompression therapy. All donors provided written informed consent. The research was conducted in accordance with the Declaration of Helsinki and received approval from the Ethics Committee of Shandong University Hospital.

### Data source and single-cell sequencing downstream analysis workflow

Bulk RNA expression data were obtained from The Cancer Genome Atlas (TCGA) and the Chinese Glioma Genome Atlas (CGGA). Single-cell RNA sequencing data for IDH-wild-type glioblastoma were sourced from the Gene Expression Omnibus (GEO; accession GSE131928), and the raw 10X Genomics files from this dataset were downloaded for further processing.

Downstream analysis was performed using Seurat (v5.0.1). Quality control removed cells with > 20% mitochondrial gene content (percent.mt), < 500 detected genes, or total RNA counts < 100. Each sample was processed individually. Expression matrices were normalized via the LogNormalize method using a scale factor of 10,000, and the top 2000 highly variable genes were selected with the FindVariableFeatures function under the "vst" setting. Data were scaled and subjected to PCA. A k-nearest neighbor graph was built using the top 20 PCs (FindNeighbors), followed by clustering (FindClusters, resolution = 0.5). UMAP was applied for visualization. Batch effects were corrected with Harmony.

For CNV analysis, inferCNV was used with cells down-sampled to 2000 per sample. A pseudo-bulk matrix was generated via AverageExpression to reduce false positives. Differential gene expression between groups was assessed using FindAllMarkers, with thresholds set at |log_2_FC| > 0.5 and adj. p-value < 0.05. Metabolic and autophagy scores were computed with AddModuleScore, using gene sets from KEGG and Reactome databases in MSigDB.

### Cell lines and culture

The study employed multiple human GBM cell lines alongside a normal human astrocyte (NHA) control. GBM#P3 (including a luciferase-stable variant), GBM#BG5, and GBM#BG7 were gifts from Prof. Rolf Bjerkvig. Additional established GBM lines like T98G, U118MG, A172, and LN229 were obtained from the National Collection of Authenticated Cell Cultures (Shanghai, China). Cells were maintained under distinct culture conditions optimized for their respective phenotypes. The patient-derived GBM#P3, BG5, and BG7 lines were grown in Neurobasal Medium. In contrast, the established GBM cell lines and NHA were cultured in DMEM supplemented with 10% fetal bovine serum (FBS). All media and reagents were sourced from Thermo Fisher Scientific or GE Healthcare Life Sciences, as indicated.

### Lactate content and LDH activity assay

Tissues were homogenized in Lactate Assay Buffer (100 μL per 10 mg), centrifuged at 12,000g for 5 min at 4 °C, and lactate levels were measured using a lactate assay kit (beyotime, S0208S) based on absorbance at 450 nm. LDH activity was determined using a beyotime kit (P0393S): tissues were ground, ultrasonicated, centrifuged at 12,000g for 10 min, and the supernatant was diluted. Absorbance was measured at 450 nm after WST-8 chromogenic reaction.

### Small molecule drugs

The key small molecule compounds utilized in this study include: BafA1 (SigmaAldrich, B1793), Rapamycin (Selleck, S1039), Sodium Oxamate (Target Mol, T19831), 3-MA (Selleck, S2767), ZZW-115 (Selleck, E1037), and Sodium lactate (Selleck, S6010). 3-MA, BafA1, rapamycin and Sodium lactate were dissolved in DMSO, while ZZW-115 Sodium Oxamate were dissolved in water. For *in vitro* experiments, these compounds were diluted with culture medium to achieve the respective working concentrations (3-MA: 10 mM; BafA1: 100 nM; Rapamycin: 100 nM; ZZW-115: 3 or 6 μM; Nala: 20 mM; Sodium Oxamate: 50 µM).

### NUPR1 silencing and overexpression

siRNAs targeting NUPR1 (si-NUPR1#1: GCUGCCAUCAACACAAUGUTT; si-NUPR1#2: GCAGAUGAAUGCUUUAUAATT) were obtained from BioSune (Shanghai). For overexpression, full-length NUPR1 cDNA was cloned into pENTER. Transfections used Lipofectamine™ 2000 (siRNA) or Lipofectamine Lipo3000 (plasmid). Stable knockdown and overexpression were achieved using lentiviral shRNAs (GenePharma). Efficiency was validated via qRT-PCR and western blot after 48 h.

### Cell counting kit-8 (CCK-8) assay

Cell viability was evaluated using the CCK-8 assay. Briefly, glioma cells were seeded into 96-well plates at a density of 2.0 × 10^4^ cells per well and cultured under standard conditions. Following treatment, cells were incubated with 10 µL of CCK-8 reagent in 100 µL serum-free DMEM for 4 h. Absorbance was then measured at 450 nm using a microplate reader.

### 5-Ethynyl-2'-deoxyuridine (EdU) incorporation assay

Proliferation was assessed via an EdU assay kit (Yeasen, 40275ES60). Cells in the logarithmic growth phase were treated with 10 µM EdU for 2 h, fixed with 4% paraformaldehyde, and permeabilized with 0.5% Triton X-100. A Click-iT reaction was performed according to the manufacturer's protocol, followed by optional nuclear counterstaining with Hoechst 33342. Fluorescence microscopy was used to visualize and quantify EdU-positive cells.

### Transwell assay

Cell invasion was examined using Matrigel-coated Transwell chambers. The bottom membrane of each insert was coated with a diluted Matrigel solution. LN229 cells (5 × 10⁴cells per insert) suspended in serum-free medium were added to the upper chamber, while the lower compartment contained medium supplemented with 30% FBS. After 36 h of incubation. Cells that had migrated to the lower membrane surface were fixed, stained with crystal violet, and quantified by light microscopy.

### Protein extraction

Total protein was isolated from tissue or cell samples with RIPA lysis buffer containing protease and phosphatase inhibitors. Following a 30 min incubation on ice, lysates were centrifuged at 12,000g for 15 min at 4 °C to collect the supernatant. Protein concentration was quantified with the BCA assay, and samples were normalized to ensure equal loading. After denaturation with loading buffer at 100 °C for 10 min, 30 µg of protein per sample was resolved on 12% SDS-polyacrylamide gels.

### Western blotting

Separated proteins were transferred onto polyvinylidene difluoride membranes via electrophoresis. The membranes were then blocked at room temperature for 2 h using 5% non-fat milk in TBST buffer, after which they were incubated with primary antibodies overnight at 4 °C under gentle shaking. Membranes were then washed three times in TBST and incubated with HRP-conjugated secondary antibodies for 2 h. Protein bands were visualized and captured using a ChemiDoc XRS+ imaging system. The following primary antibodies were showed in Table S1.

### 3D tumor sphere invasion assay

To assess invasive potential, GBM#P3 cells were seeded at a density of 5×10³ per well in low-adhesion plates to facilitate tumor sphere formation. Following sphere establishment, invasion gel (R&D Systems, #3500-096-03) was introduced, and the invasive capacity of the spheres was tracked for a period of 72 h.

### Wound healing assay

LN229 cells were first cultured to full confluence in 6-well plates. A scratch was made with a pipette tip. Dislodged cells were cleared through two washes with PBS, followed by the addition of serum-free medium, and migration was imaged at 0, 48, and 96 h. The relative wound closure rate was quantified using ImageJ software by measuring the remaining scratch area at each time point.

### Transmission electron microscopy

Following treatment, cells were trypsinized and pelleted, then sequentially fixed with 2.5% glutaraldehyde and post-fixed with 1% osmium tetroxide, followed by uranyl acetate staining, ethanol gradient dehydration, and epoxy resin embedding for ultrastructural analysis. Ultrastructure was imaged with a JEM-1200EX II microscope.

### Immunofluorescence

The prepared frozen sections were washed with PBS after returning to room temperature, blocked for 1 hour using immunofluorescence blocking buffer (containing 0.5% Triton), and then incubated with anti-H4K8la (PTM-1415, PTM BIO, 1:200), anti-NUPR1 (PA1-4177, Thermo Fisher Scientific, 1:100) and anti-Lactyl Lysine (PTM-1401RM, PTM BIO, 1:200) in 5% bovine serum albumin (BOSTER, AR0004) in PBS overnight. Primary antibody was detected with Goat Anti-Mouse IgG H&L (Alexa Fluor® 488) (Abcam, ab150177) and Goat Anti-Rabbit IgG H&L (Alexa Fluor® 594) (Abcam, ab150088). Cells were incubated in the dark with DAPI to stain nuclei. Fluorescence microscopy was performed to examine the slides, and digital images were captured with an Olympus DP71 charge-coupled device camera (Waltham, MA, USA).

### Tissue processing and immunohistochemistry

Tissue samples embedded in paraffin were sectioned into 5 µm slices and mounted on glass slides. Antigen retrieval was conducted by microwave heating in EDTA-based retrieval buffer (Solarbio). Subsequently, sections were incubated with the primary antibody (anti-NUPR1 PA1-4177, Thermo Fisher Scientific, 1:100; anti-Ki67, 9027, Cell Signaling Technology, 1:200; anti-Lactyl Lysine, PTM-1401RM, PTM BIO, 1:400; anti-H4K8la, PTM-1415, PTM BIO, 1:200; anti-LC3B 43566S, Cell Signaling Technology, 1:200; anti-SQSTM1/p62, 39749, Cell Signaling Technology, 1:200) at 4 °C for more than 12 h. After rinsing with PBS, sections were incubated with horseradish peroxidase-conjugated goat anti-rabbit secondary antibody (ZSGB-BIO, PV-9000). Color development was performed using diaminobenzidine (ZSGB-BIO, ZLI-9033) as the substrate, followed by counterstaining with Mayer's hematoxylin (Beyotime Biotechnology, C0107).

### mCherry-EGFP-LC3B adenoviral transduction

The mCherry-EGFP-LC3B adenoviral vector (C3013, Beyotime, China) was utilized according to the manufacturer's protocol. LN229 and GBM#P3 cells were seeded in 96-well plates and transduced at varying MOI values. Transduction efficiency was monitored by fluorescence microscopy at designated time points. Following stable expression, treated cells were visualized via confocal microscopy for further analysis.

### Co-immunoprecipitation (Co-IP)

Co-IP was conducted using the Pierce™ Classic Magnetic IP/Co-IP Kit (Thermo Fisher Scientific, 88804) in strict accordance with the manufacturer's protocol. The cell lysates were first incubated overnight at 4 °C with antibodies against NUPR1 (15056-1-AP, Proteintech), P300 (Santa Cruz Biotechnology, SC-48343), HDAC2 (Santa Cruz Biotechnology, SC-9959), VPS33A (Proteintech, 16896-1-AP), H4K8la (PTM-1415, PTM BIO, 1:1000) or control IgG (Rabbit IgG: 30000-0-AP, Proteintech; Mouse IgG, B900620, Proteintech). Subsequently, Protein A/G magnetic beads (88804, Thermo Fisher Scientific) were added to the mixtures, followed by a 1 h incubation. Following elution, the proteins were subjected to SDS‒PAGE separation and subsequent western blot analysis.

### Mass spectrometry (MS)

Mass spectrometry analysis was performed on co-immunoprecipitated proteins. Briefly, the samples were separated via SDS-PAGE. The protein bands were excised for in-gel digestion, followed by MS-based identification of protein composition. The MS analysis was carried out by Novogene Co., Ltd.

### Chromatin immunoprecipitation (ChIP) and ChIP-qPCR

GBM#P3 and LN229 cells were fixed via cross-linking with a 10-minute cross-linking procedure using 1% formaldehyde, after which the reaction was terminated by adding glycine (1.25 mol/L), washed, and lysed. Chromatin was sonicated to ~300 bp, centrifuged, and diluted in IP buffer. Samples were incubated overnight at 4 °C with anti-H4K8la or IgG antibody, prior to a 4-hour binding with Protein G beads. After washing, complexes were de-cross-linked, and DNA was purified. qPCR was conducted on a QuantStudio5 platform using SYBR Green Master Mix, with specific primer sequences of NUPR1 provided in Supplementary Table S2.

### CUT&Tag

CUT&Tag assays were performed using the Hyperactive *In Situ* ChIP Library Prep Kit for Illumina (pG-Tn5) following the manufacturer's protocol. Briefly, GBM#P3 cells were immobilized using concanavalin A-coated magnetic beads. The cells were then resuspended in antibody buffer and incubated sequentially with a primary antibody targeting H4K8la, followed by a secondary antibody. Subsequently, the samples were combined with protein A-Tn5 transposase and co-incubated. Tagmentation was initiated to fragment the DNA and incorporate sequencing adapters. The resulting DNA fragments were then amplified and purified to construct the sequencing library. Purification was carried out using VAHTS DNA Clean Beads. Library quantification was performed with the VAHTS Library Quantification Kit for Illumina, and paired-end sequencing was conducted on an Illumina NovaSeq platform with 150 bp read length.

### Glioma intracranial xenograft model

Male athymic mice (28 days, 22-25g) were obtained from Charles River Laboratories (CRL) (Beijing, China). Prior to intracranial tumor implantation, mice were placed under anesthesia and securely positioned within a stereotactic apparatus. A midline scalp incision was made, followed by the drilling of a 1 mm burr hole 2.5 mm lateral to the bregma. 2 × 10⁵ luciferase-expressing GBM#P3 glioma cells suspended in 20 μL of serum-free DMEM were stereotactically injected into the right striatum at a depth of 2.5 mm with a Hamilton syringe. Bioluminescent signals were monitored weekly using an IVIS-200 cooled charge-coupled-device camera (Xenogen, Alameda, CA, USA) and quantified with Living Image 2.5 software (Xenogen).

### Quantification of ZZW-115 by liquid chromatography-tandem mass spectrometry (LC-MS)

The concentration of ZZW-115 in tissue samples was determined using LC-MS. Chromatographic separation was performed on an Agela Venusil C18 Plus column (50 × 2.1 mm, 5 μm) with a gradient of 0.1% formic acid in water and methanol. Mass detection was carried out in positive electrospray ionization mode with selected reaction monitoring (SRM). A standard curve was constructed in the range of 0.2-1000 ng/mL using weighted linear regression. Tissue samples were homogenized in methanol, centrifuged, filtered, and analyzed against the standard curve. The detection technology was provided with the assistance of Servicebio Company.

### Ethics approval and conduct of experiments

All procedures involving animals in this research received ethical approval from the Committee of Qilu Hospital (Approval No. DWLL-2021-154). All experiments were strictly conducted in accordance with the approved protocol, the ARRIVE guidelines, and relevant national or institutional regulations concerning animal care and use. The maximum allowed tumor burden as defined in the protocol approved by the Ethics Committee for this study was: monitoring via the small animal *in vivo* imaging system, the experimental endpoint was reached when the fluorescence signal intensity reached a pre-set threshold, or when animals exhibited any predefined neurological deficits affecting their welfare or a weight loss exceeding 20% as an early humane endpoint indicator. We hereby confirm that none of the experiments in this study exceeded the maximum tumor burden permitted by the aforementioned Ethics Committee.

### Statistical analysis

Continuous data are expressed as mean ± standard deviation (SD). For comparisons between two groups, a paired Student's t-test was applied. Comparisons among three or more groups were performed using one-way ANOVA, with appropriate post-hoc correction for multiple comparisons to control the false discovery rate. Survival analysis was performed with the Kaplan-Meier method, and between-group differences were assessed using the log-rank test. Statistical significance was defined as *P < 0.05, **P < 0.01, and ***P < 0.001.

## Results

### Elevated levels of histone lactylation correlate with poor clinical outcomes in glioma patients

**Figure 1:**
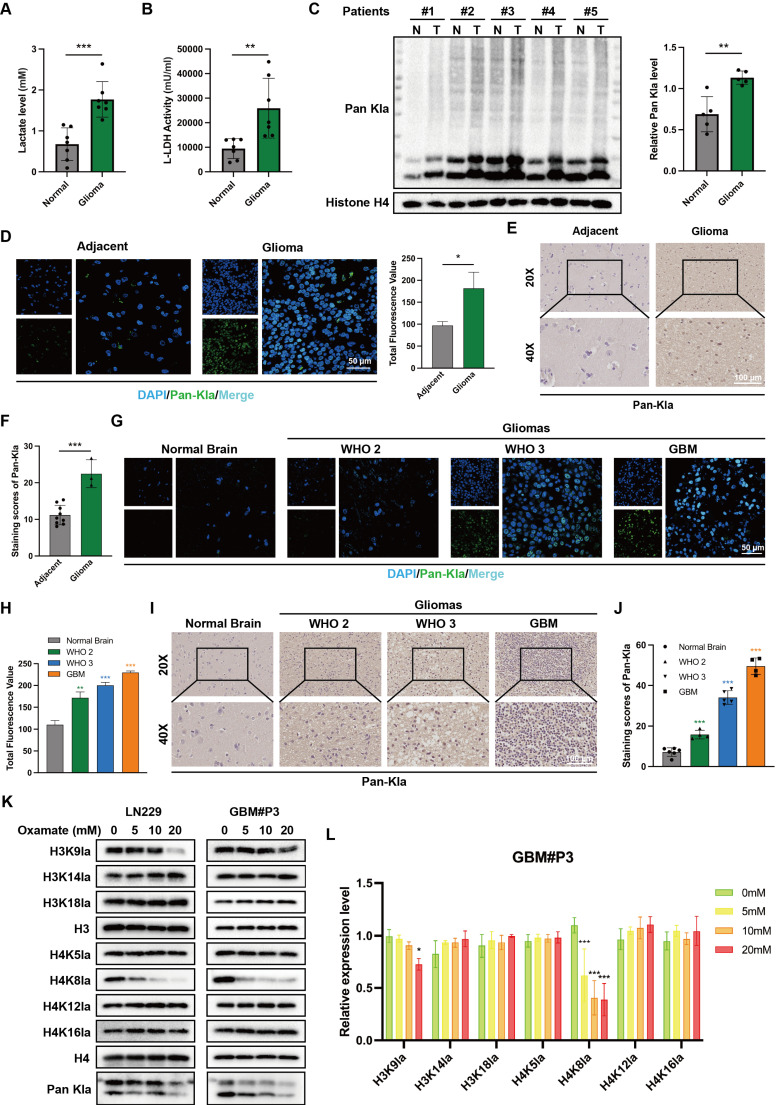
** Histone lactylation correlates with malignant progression and unfavorable prognosis in glioma.** (A) Comparison of lactate content in tissues from glioblastoma patients and paired normal brain tissue donors. (B) Comparison of LDH levels between tumor tissues and normal brain tissues from glioma patients. (C) Panlysine lactylation (pan-Kla) levels in paired GBM tissue and adjacent normal brain tissues were measured by Western blotting with quantification of grayscale values. (D) IF staining and statistical results of lactylation in adjacent tumor tissue and glioma tissues (scale bars: 50 μm). (E, F) IHCstaining results and statistical analysis of pan-Kla expression in GBM tumor tissues and adjacent tissues (scale bars: 100 μm). (G, H) IF staining and statistical graphs showing pan-Kla levels and subcellular localization in NBT and gliomas of different grades (scale bars: 50 μm). (I, J) IHC staining images and corresponding quantification scores for pan-Kla in gliomas across different grades (scale bars: 100 μm). (K) Western blot analysis of lactylation levels at different specific histone lysine sites in LN229 and GBM#P3 glioma cells. (L) Quantification of grayscale values of lactylation levels at different specific histone lysine sites. Data are presented as mean ± SD. Significance levels between specified treatment groups are indicated as follows: *P < 0.05, **P < 0.01, ***P < 0.001.

Initial analysis detected the lactate content in tumor tissues from glioma patients and normal brain tissues from donors. The results revealed a significantly higher lactate concentration in glioma tissues than in normal brain tissues (NBT) with unpaired t-tests (Figure 1A). Concurrent assessment of lactate dehydrogenase (LDH) activity in these tissues demonstrated that LDH levels were also elevated in tumor samples (Figure 1B). These findings indicate both an accumulation of lactate and highly activated lactate metabolism in glioma. Current research suggests that the accumulation of substantial amounts of lactate provides abundant substrate for protein lactylation. Accordingly, the levels of global protein lactylation were examined in ten samples of GBM tissue and adjacent non-tumor tissue. Western blot analysis and grayscale quantification revealed a significant increase in panlysine lactylation (pan-Kla) levels in GBM tissues compared with those in adjacent tissues (Figure 1C and Figure S1A). Additionally, lactylated proteins were predominantly concentrated within the 17-8 kDa range, indicating the potential presence of substantial histone lactylation modifications.

Immunofluorescence (IF) staining was subsequently performed to further compare the differential lactylation levels between tumor and adjacent tissues in glioma samples and to identify regions with widespread lactylation modifications (Figure 1D). The results indicated markedly higher panlactylation levels in tumor tissues than in normal tissues. Furthermore, the fluorescent signals predominantly overlapped with the nuclear staining, suggesting the occurrence of histone lactylation modifications. This conclusion was further supported by subsequent immunohistochemical (IHC) results (Figure 1E-F). Protein extracts were subsequently prepared from human normal glial cells (NHA) and multiple glioma cell lines and primary cells, including T98G, U118MG, A172, LN229, GBM#P3, GBM#BG5, and GBM#BG7. Pan-lactylation levels were assessed by Western blot analysis. The results revealed elevated pan-Kla levels in most glioma cell lines, with particularly pronounced increases observed in LN229 and GBM#P3 cells (Figure S1B).

To investigate the correlation between pan-Kla levels and the prognosis of glioma patients, IF (Figure 1G-H) and IHC staining (Figure 1I-J) were performed on tissue sections from patients with different glioma grades. The results indicated that pan-Kla levels increased with increasing glioma grade, with more pronounced nuclear localization observed in GBM. In summary, our preliminary data suggest that elevated histone lactylation levels may indicate higher glioma grade and worse prognosis.

### Altered lactylation at histone H4K8 plays a significant role in glioma progression

**Figure 2:**
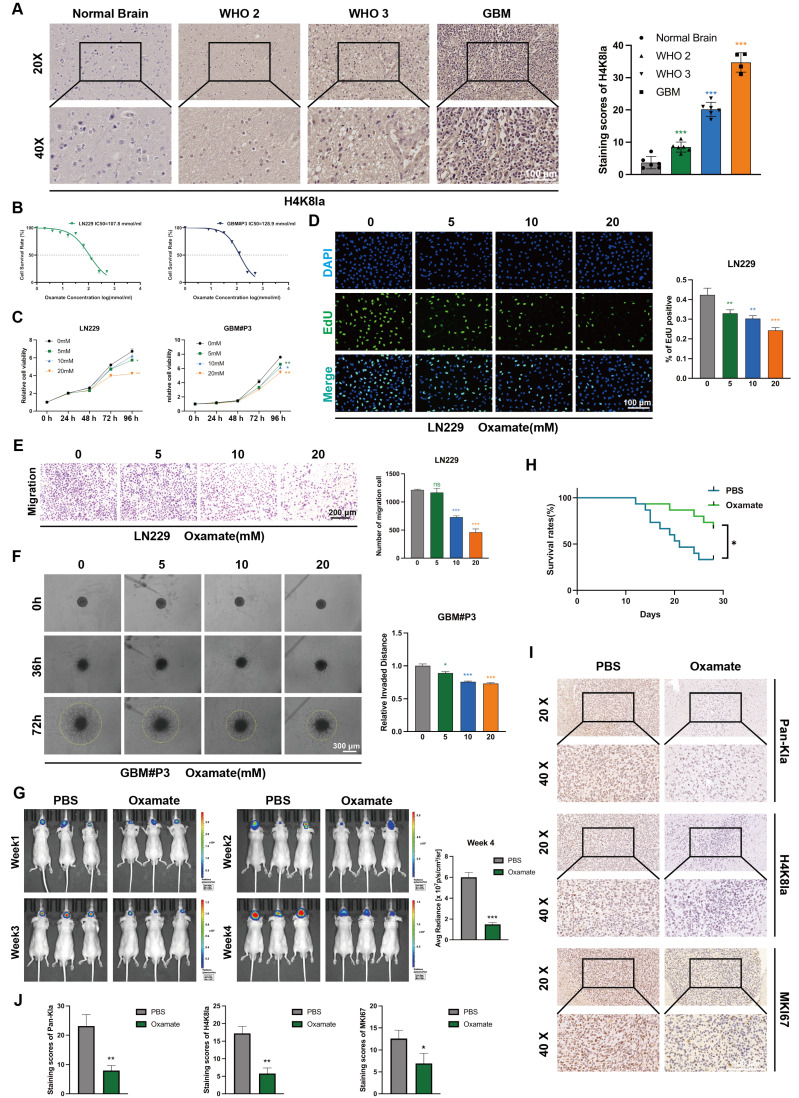
** Glycolysis inhibition reduces H4K8la and suppresses GBM progression *in vitro* and *in vivo*.** (A) Representative IHC staining images for H4K8la in NBT and gliomas of different grades and IHC staining scores for H4K8la in NBT and in gliomas of different histological grades (scale bars: 100 μm). (B) IC50 curves of the glycolytic inhibitor oxamate in LN229 and GBM#P3 cells. (C) A CCK8 assay was used to analyse the effect of oxamate on LN229 and GBM#P3 cell viability. (D) The effects of different concentrations of oxamate on the proliferation of glioma cells were determined via an EdU staining assay (scale bar: 100 μm). (E) Transwell invasion assays showing the migration capacity of LN229 cells treated with different concentrations of oxamate (scale bars: 200 μm). Representative images and quantitative analysis. (F) 3D invasion assays evaluating the penetration ability of GBM#P3 cells treated with oxamate with quantitative analysis (scale bars: 300 μm). (G) Luciferase-expressing GBM#P3 cells were orthotopically implanted into athymic nude mice. Tumor growth was monitored by detecting bioluminescence weekly using an IVIS-200 imaging system, with luminescence values quantified at week 4. (H) Overall survival was determined using Kaplan-Meier survival curves, and a log-rank test was used to assess the statistical significance of the differences. (I, J) Images and corresponding quantitative analysis of IHC staining for pan-Kla, MKI67 and H4K8la in tumors from the indicated groups. (scale bars: 100 μm). Data are presented as mean ± SD. Significance levels between specified treatment groups are indicated as follows: *P < 0.05, **P < 0.01, ***P < 0.001.

To further evaluate the histone lactylation site with the most prominent changes during glioma progression, we examined the lactylation levels of 7 histone sites in GBM#P3 and LN229 cells via Western blotting (Figure 1K). We subsequently quantified the grayscale values to assess the most dynamically altered histone lactylation sites, confirming that both H3K18la and H4K8la were modified, with H4K8la displaying the most pronounced changes (Figure 1L). The heatmap further illustrates differential lactylation patterns across various histone sites (Figure S1C). IHC analysis revealed that, compared with NBT, H4K8la expression was significantly upregulated with increasing glioma grade (Figure 2A). These findings are consistent with conclusions from previous studies. Moreover, the IHC results for both adjacent nontumor tissues and tumor tissues indicated that compared with that in adjacent nontumor tissues, the level of H4K8la in tumors was significantly upregulated (Figure S2A).

### Inhibition of glycolysis decreases histone lactylation levels and impairs the proliferation, invasion, and migratory capacity of GBM cells

To investigate the role of histone lactylation in promoting GBM progression *in vitro* and further elucidate the influence of glycolysis on histone lactylation, GBM cells were treated with the glycolytic inhibitor oxamate to reduce lactylation levels. We first predicted the tolerance of GBM cells to oxamate and the appropriate dosage by plotting IC50 curves (Figure 2B). We evaluated the viability of GBM cells treated with 5, 10 and 20 mM oxamate using CCK-8 assays. The results demonstrated that oxamate effectively reduced the viability of both GBM#P3 and LN229 cells, with 20 mM oxamate having the most potent inhibitory effect on cell viability (Figure 2C). Consistently, cell proliferation assessed by EdU incorporation was markedly inhibited by oxamate treatment in GBM cells (Figure 2D).

To explore the effects of oxamate on the invasive and migratory properties of GBM cells, the distant invasion capacity of LN229 cells under varying oxamate concentrations was evaluated using Transwell chamber assays (Figure 2E). We also added Matrigel to the Transwell chambers to validate the effect of oxamate on the invasiveness of LN229 cells. The results indicate that oxamate effectively inhibits the invasive ability of glioma cells (Figure S2B). Additionally, a 3D invasion assay was conducted to evaluate the ability of GBM#P3 primary cells to penetrate Matrigel and achieve distant dissemination (Figure 2F), which revealed that higher concentrations of oxamate markedly reduced the invasive range of these primary GBM cells. Furthermore, scratch wound healing assays confirmed that oxamate treatment significantly inhibited the distant migration and repair capacity of LN229 cells (Figure S2C). In summary, *in vitro* experiments preliminarily demonstrate that lactylation of histone plays a critical role in pathogenesis and progression of GBM, suggesting that targeting this modification may hold potential therapeutic value against tumors.

We subsequently divided nude mice into two groups. With the assistance of a stereotaxic instrument, we established orthotopic tumors using luciferase-stably labelled GBM#P3 cells. The mice then received intraperitoneal injections of either oxamate or an equal volume of PBS. The tumor luminescence signals were monitored continuously for four weeks (Figure 2G). Simultaneously, a Kaplan-Meier survival curve was plotted based on the survival data of the mice (Figure 2H). Upon completion of the four-week period, surviving mice were euthanized, after which brain tissues were collected, fixed in paraffin, and sectioned for subsequent analysis. IHC staining was performed for three markers: KI67, pan-Kla, and H4K8la (Figure 2I, J). The results demonstrated that compared with the control group, the oxamate-treated group exhibited slower tumor growth and prolonged survival. Consistent with the growth inhibition observed, immunohistochemical analysis demonstrated reduced expression of the proliferation marker MKI67 in oxamate-treated tumors, while the expression of pan-Kla and H4K8la was also decreased.

### Exogenous lactate supplementation can reverse the downregulation of lactylation levels induced by glycolysis inhibition

**Figure 3:**
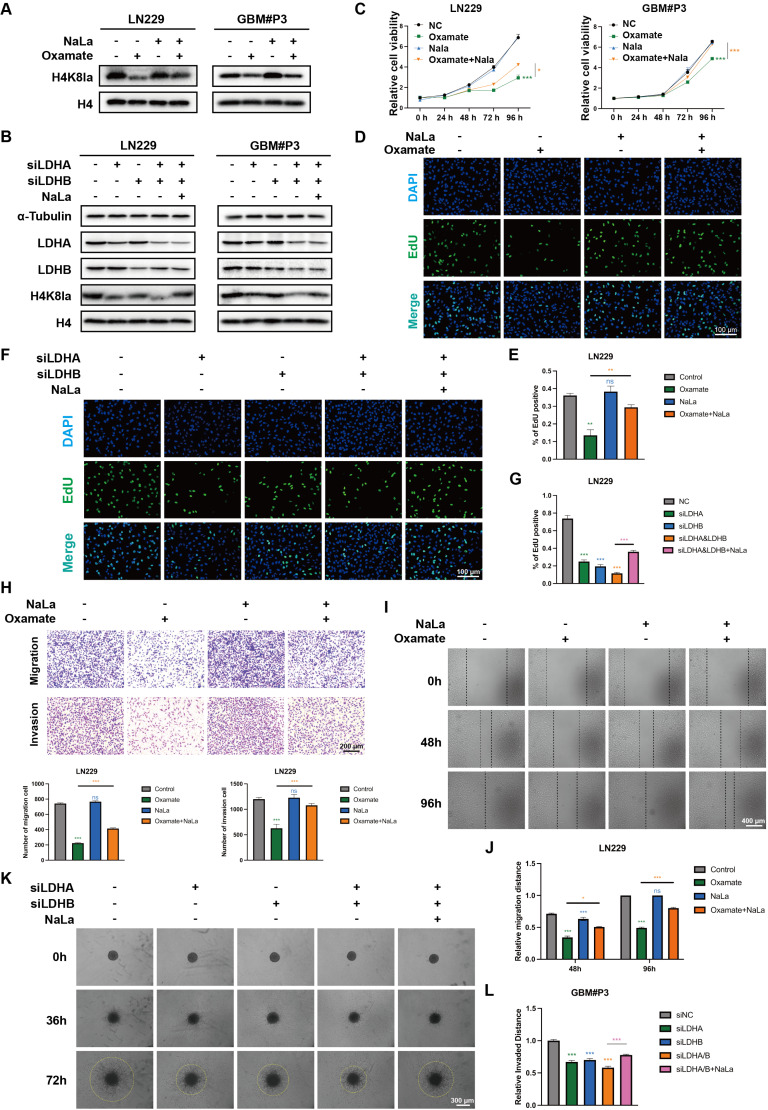
** Exogenous lactate supplementation reverses the downregulation of lactylation levels induced by glycolysis inhibition and affects the malignant phenotype of GBM.** (A) Western blot results demonstrating alterations in H4K8la levels following oxamate and NaLa treatment. (B) Western blot results demonstrating alterations in H4K8la levels after the knockdown of LDHA or/and LDHB and NaLa treatment. (C) CCK8 assays were used to analyse cell viability after LDHA and/or LDHB and Nala knockdown. (D, E) Representative micrographs and quantitative assessment of the proliferation ability of Nala- and oxamate-treated LN229 cells after incubation via an EdU staining assay (scale bars: 100 μm). (F, G) Representative images and statistical analysis of the proliferation ability of siLDHA/siLDHB-treated LN229 and GBM#P3 cells after Nala incubation were analysed via an EdU staining assay (scale bars: 100 μm). (H) Representative images and quantitative analysis of the Transwell invasion assays showing the invasive capacity of LN229 cells treated with oxamate and Nala (scale bars: 200 μm). (I, J) Representative images and quantitative analysis of wound-healing assays showing the tumor migration ability of LN229 cells treated with oxamate and Nala (scale bars: 400 μm). (K, L) 3D invasive spheroid assay images and statistical analysis demonstrating the distant invasive capacity of GBM#P3 cells supplemented with Nala following siLDHA and/or siLDHB treatment (scale bars: 300 μm). Data are presented as mean ± SD. Significance levels between specified treatment groups are indicated as follows: *P < 0.05, **P < 0.01, ***P < 0.001.

To further clarify whether the phenotypic changes in GBM induced by oxamate are associated with alterations in lactylation, we conducted a rescue experiment by supplementation with exogenous sodium lactate (NaLa). Western blot results revealed that H4K8la levels decreased with increasing inhibitor concentration but were restored upon NaLa supplementation (Figure 3A). LDHA and LDHB are key enzymes in lactate metabolism, and inhibiting their expression strongly suppresses the Warburg effect. We designed small interfering RNAs (siRNAs) to suppress LDHA, LDHB, or both in LN229 and GBM#P3 cells. Western blot analysis confirmed that siLDHA partially reduced H4K8la levels, whereas NaLa supplementation reversed this reduction (Figure 3B). Subsequently, the functional consequences of histone lactylation modulation on cellular viability and proliferation were examined. CCK-8 assays demonstrated that NaLa supplementation could reverse the oxamate-induced decrease in cell viability (Figure 3C), while EdU proliferation assays confirmed that NaLa could reverse the decline in cell proliferative capacity (Figure 3D-E). Similarly, when siLDHA and/or siLDHB were applied, the subsequent addition of NaLa also rescued the observed reductions in both cell viability and proliferation (Figure 3F-G and Figure S3A).

In addition, Transwell assays revealed that oxamate treatment or LDH knockdown impaired the migration and invasive capacity of GBM cells, whereas NaLa-supplemented tumor cells exhibited enhanced distant invasion (Figure 3H and Figure S3B). Wound-healing assays demonstrated that glycolysis inhibitors and siLDHA/LDHB partially reduced the migratory repair ability of LN229 cells (Figure 3I-J and Figure S3C), while exogenous lactate supplementation promoted tumor migration. Finally, 3D invasion assays confirmed that oxamate treatment and LDHA/LDHB knockdown inhibited the spatial invasive ability of primary GBM#P3 cells, and this suppression was reversed by sodium lactate supplementation (Figure 3K-L and Figure S3D).

In previous studies, modifying enzymes have been considered important regulatory factors of histone lactylation. We found that 9 enzyme genes, previously identified as having potential lactylation regulatory capabilities (Table S3), showed significant alterations following RNA sequencing via normal and glioma patients (Figure S3E). In our CUT&Tag results, we found that the levels of acetyltransferase P300 and HDAC2 are associated with the levels of histone lactylation 37. P300 has been documented in existing literature as a potential writer of histone lactylation in pancreatic ductal adenocarcinoma 38. Meanwhile, HDAC2, a key member of the histone deacetylase (HDACs) family, has been reported to regulate histone lactylation modifications at specific sites during angiogenesis and bladder cancer progression 39, 40. Therefore, we validated the potential physical interactions between H4K8la and P300/HDAC2 through co-immunoprecipitation (Co-IP) experiments. We found that immunoprecipitation using an anti-H4K8la antibody successfully co-precipitated both P300 and HDAC2, which was further confirmed by Western blot analysis ((Figure S3F.). Reciprocal experiments yielded consistent results. These findings are consistent with previous studies and provide strong evidence for the functional correlation between this regulatory enzyme system and histone modification sites.

### H4K8la activates NUPR1 transcription in GBM cells

**Figure 4:**
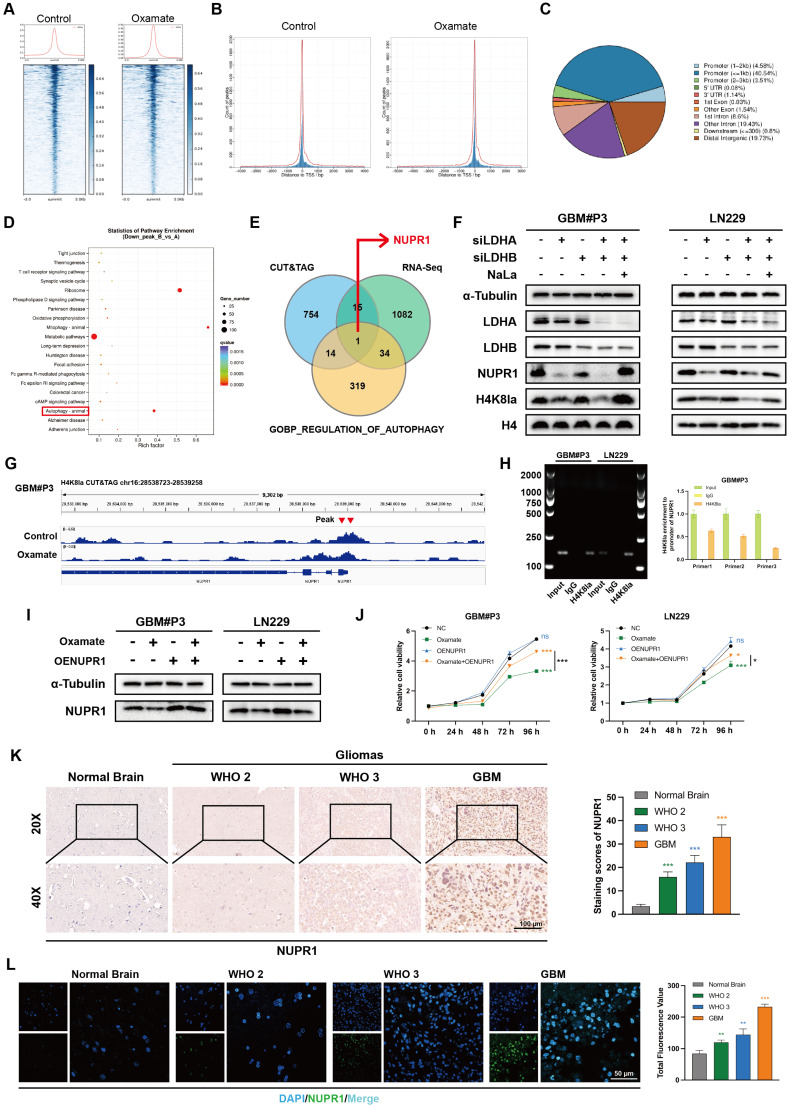
** H4K8la directly binds to the NUPR1 promoter and activates its transcription.** (A-C) CUT&Tag analysis using an anti-H4K8la antibody revealed a significant reduction in H4K8la enrichment at transcription start sites (TSSs) and promoter regions in oxamate-treated GBM cells. (D) KEGG pathway analysis of genes whose H4K8la promoter occupancy was reduced in the oxamate-treated group. (E) Venn diagram showing that NUPR1, a key autophagy-related gene, is downregulated by oxamate and bound by H4K8la. (F) Western blot analysis showing changes in NUPR1, LDHA, LDHB, and H4K8la levels in GBM cells after LDHA/LDHB knockdown and NaLa rescue treatment. (G) Integrative Genomics Viewer tracks of CUT&Tag showing enriched H4K8la in the promoters of NUPR1. (H) DNA fragments were immunoprecipitated with the H4K8la antibody and analysed by gel electrophoresis and qPCR. (I) Western blot showing alterations in NUPR1 expression in GBM cells following oxamate treatment and oeNUPR1 transfection. (J) CCK-8 assay revealing changes of GBM#P3 and LN229 cells after oxamate treatment and oeNUPR1 transfection in the cell viability. (K) IHC images and statistical analysis showing NUPR1 expression in gliomas of different grades and NBT. (L) IF staining and statistical graphs showing NUPR1 expression and subcellular localization in NBT and gliomas of different grades. Data are presented as mean ± SD. Significance levels between specified treatment groups are indicated as follows: *P < 0.05, **P < 0.01, ***P < 0.001.

Next, we sought to determine how H4K8la influences GBM progression. Based on existing evidence that histone lactylation functions as a direct epigenetic regulator of chromatin-dependent transcription, CUT&Tag assays were conducted with an anti-H4K8la antibody. Analysis showed a marked decrease in signal intensity at transcription start sites (TSS) upon oxamate treatment (Figure 4A-B), while promoter regions exhibited approximately 49% enrichment under the same condition (Figure 4C).

To further identify potential target genes regulated by lactylation, RNA sequencing (RNA-seq) analysis was performed. A volcano plot indicated that compared with control treatment, oxamate treatment upregulated 869 genes and downregulated 1132 genes in GBM#P3 cells (Figure S4A). KEGG pathway analysis further indicated that the set of downregulated genes was enriched in autophagy-related pathways following oxamate treatment (Figure 4D). We then generated a Venn diagram to identify genes that were differentially expressed between the CUT&Tag and RNA-seq analyses and key autophagy-related genes (Figure 4E), ultimately identifying NUPR1 as a critical autophagy-related gene. Western blot results confirmed that siLDHA and siLDHB significantly reduced the protein expression level of NUPR1 together with H4K8la (Figure 4F). We found that the peak at the NUPR1 promoter site was significantly reduced in oxamate-treated cells (Figure 4G). Using ChIP‒qPCR assays, we further confirmed that H4K8la was enriched at the NUPR1 promoter and was reduced by oxamate treatment (Figure 4H and Figure S4B).

To further confirm the influence of lactate metabolism on NUPR1 expression, Western blot analysis was performed in LN229 and GBM#P3 cells following oxamate treatment and NUPR1 overexpression (Figure 4I). The results indicated that the expression of NUPR1 decreased with the addition of oxamate and re-overexpression of NUPR1 partially reversed the oxamate-induced downregulation of NUPR1. Moreover, CCK8 assays confirmed that the oxamate-induced decrease in cell viability could be reversed by overexpressing NUPR1 (Figure 4J). To further evaluate NUPR1 expression in glioma patients, we performed IHC staining. The results revealed that in NTB tissues, NUPR1 expression was relatively low and was predominantly localized in the cytoplasm. However, as the glioma grade increased, NUPR1 expression progressively increased, and NUPR1 accumulated in the nucleus (Figure 4K). Consistent findings were obtained through IF assays (Figure 4L). Combined with data from the TCGA database, we performed survival analysis on GBM patients with varying NUPR1 expression levels. These data demonstrate that elevated NUPR1 expression correlates with worse clinical outcomes in glioblastoma patients (Figure S4C). To further evaluate the relationship between NUPR1 and glioma prognosis, we integrated clinical data and RNA sequencing results from 325 glioma patients of different grades in the CGGA database. Prognostic analysis revealed that NUPR1 exhibited the highest expression level in patients with Grade IV (Figure S4D), while the K-M curve confirmed that patients in the high NUPR1 expression group had shorter overall survival (Figure S4E). IF and IHC staining revealed significantly lower NUPR1 expression in the peritumoral region than in the tumor centre (Figure S4F-G), suggesting that NUPR1 expression is negatively correlated with glioma prognosis.

### NUPR1 is an oncogene that promotes malignant progression of gliomas

**Figure 5:**
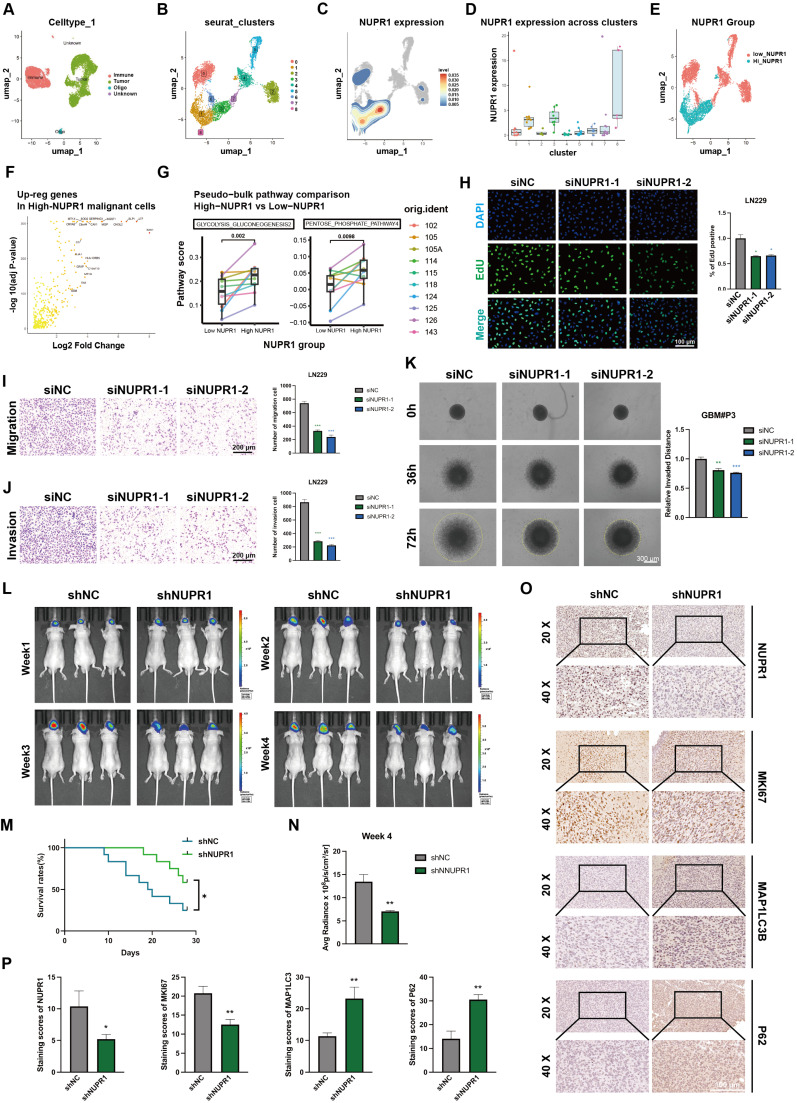
** NUPR1 expression is regulated by lactylation and promotes GBM malignancy.** (A) The UMAP visualization delineates the heterogeneous cellular composition of glioma tissue. (B)The UMAP plot illustrates the nine identified distinct tumor cell clusters. (C, D) UMAP and bar plot display the expression level of NUPR1 in malignant cell clusters. (E) Malignant cells were divided into Low NUPR1 expression cells (Low-NUPR1) and High NUPR1 expression cells (High-NUPR1). (F) The volcano plot displays the upregulated genes in High-NUPR1 cells and labels the top 20 genes with the highest absolute log2 fold change. (G) KEGG pathway activity scores demonstrate differences in metabolic pathways between glioma cells with high and low NUPR1 expression levels. (H) The effects of NUPR1 knockdown on the proliferation of LN229 cells were determined via an EdU staining assay with images and quantitative analysis (scale bars: 100 μm). (I, J) Representative images and quantitative analysis of the Transwell invasion assays showing the migration and invasive capacity of LN229 cells treated with siNUPR1 (scale bars: 200 μm). (K) 3D invasive spheroid assay results demonstrating the distant invasive capacity of GBM#P3 cells after siNUPR1 treatment (scale bars: 300 μm). (L) Following orthotopic implantation of luciferase-GBM#P3 cells into athymic nude mice, we monitored tumor growth by bioluminescence detection using an IVIS-200 imaging system. (M) Overall survival in the shNC and shNUPR1 groups was determined using Kaplan-Meier survival curves. (N) Bioluminescence values for assessing tumor growth in week 4. (O, P) Representative IHC images and corresponding quantitative analysis of NUPR1, MKI67, p62, and LC3B expression in tumor tissues across the treatment groups (scale bars: 100 μm). Data are presented as mean ± SD. Significance levels between specified treatment groups are indicated as follows: *P < 0.05, **P < 0.01, ***P < 0.001.

To further evaluate the expression pattern of NUPR1 in the progression of GBM and its relationship with metabolic reprogramming and autophagy, we analyzed single-cell RNA sequencing data from the GSE131928 dataset. After removing low-quality cells, we used Harmony to correct for batch effects across samples. PCA plots demonstrated the effective removal of batch effects (Figure 5A; Figure S4H), we performed an initial assessment of cell distribution via UMAP projection (Figure S4I-J). Tumor cells were preliminarily distinguished from immune cells based on the expression levels of PTPRC and MBP (Figure 5A; Figure S4K), malignant clusters were identified based on the evaluation of copy number variation (CNV) (Figure S4L-M).

Subsequently, we identified subpopulations within the tumor tissue through UMAP and annotated clusters 1, 3, and 8 as High-NUPR1 expression groups (Figure 5B-D). Based on the NUPR1's expression level in different groups, we generated a UMAP plot distinguishing tumor cells with high and low NUPR1 expression (Figure 5E). Differential expression analysis via volcano plot identified key upregulated genes in High-NUPR1 cells (Figure 5F). Using KEGG pathway scoring generated by AddModuleScore function, we compared metabolic pathways between GBM cells with different NUPR1 expression levels. The results indicated that cells with high NUPR1 expression were associated with enhanced glycolysis and activation of the pentose phosphate pathway (Figure 5G). Meanwhile, in tumor cells with high expression of NUPR1, the level of the tricarboxylic acid (TCA) cycle is relatively low (Figure S5A).

To further investigate the role of NUPR1 in GBM progression, we designed siRNAs to suppress NUPR1 expression in GBM cells. Western blot analysis confirmed the knockdown efficiency of the two distinct siRNAs (Figure S5B). Consistent with prior observations, CCK-8 viability assays confirmed that NUPR1 knockdown markedly reduced cellular viability in GBM cells (Figure S5C). EdU proliferation assays revealed that NUPR1 knockdown markedly suppressed the proliferative capacity of GBM cells (Figure 5H). To comprehensively evaluate the impact of NUPR1 on GBM cell invasion and migration, Transwell assays were performed with or without Matrigel coating to determine the correlation between NUPR1 expression levels and the invasive and migratory potential of LN229 cells (Figure 5I-J). The results showed that NUPR1 depletion significantly attenuated cellular invasion. Wound healing assays provided additional evidence that siNUPR1 treatment effectively inhibited both long-distance migration and wound repair capacity in LN229 cells (Figure S5D). 3-D invasion assays demonstrated that GBM#P3 cells with reduced NUPR1 expression exhibited a substantially decreased ability to penetrate Matrigel and achieve distal dissemination (Figure 5K).

Subsequent *in vivo* experiments were conducted to validate the oncogenic function of NUPR1. We generated GBM#P3 cells with stable NUPR1 knockdown using lentiviral infection and the same targeting sequence as that used for siNUPR1-1. The knockdown efficiency of shNUPR1 is shown in Figure S5E. Nude mice were divided into three groups and subjected to stereotaxic implantation of luciferase-labelled GBM#P3 cells with either stable NUPR1 knockdown (shNUPR1) or control vector (shNC). Tumor progression was tracked through weekly bioluminescence imaging over a four-week period (Figure 5L). KM survival curves confirmed that mice in the shNUPR1 group exhibited a more favourable prognosis (Figure 5M). The average radiance of the different groups in week 4 is shown in Figure 5N. At the study endpoint, the brain tissues were harvested for paraffin embedding and sectioning, followed by IHCstaining for NUPR1, KI67, LC3B and p62. Compared with the shNC group, the shNUPR1 group had significantly slower tumor growth and longer survival. IHC analysis confirmed effective NUPR1 knockdown in the shNUPR1 group, which was accompanied by decreased MKI67 expression and increased LC3B and p62 levels compared with control group (Figure 5O-P).

### NUPR1 knockdown leads to the accumulation of autophagosomes in GBM cells

**Figure 6:**
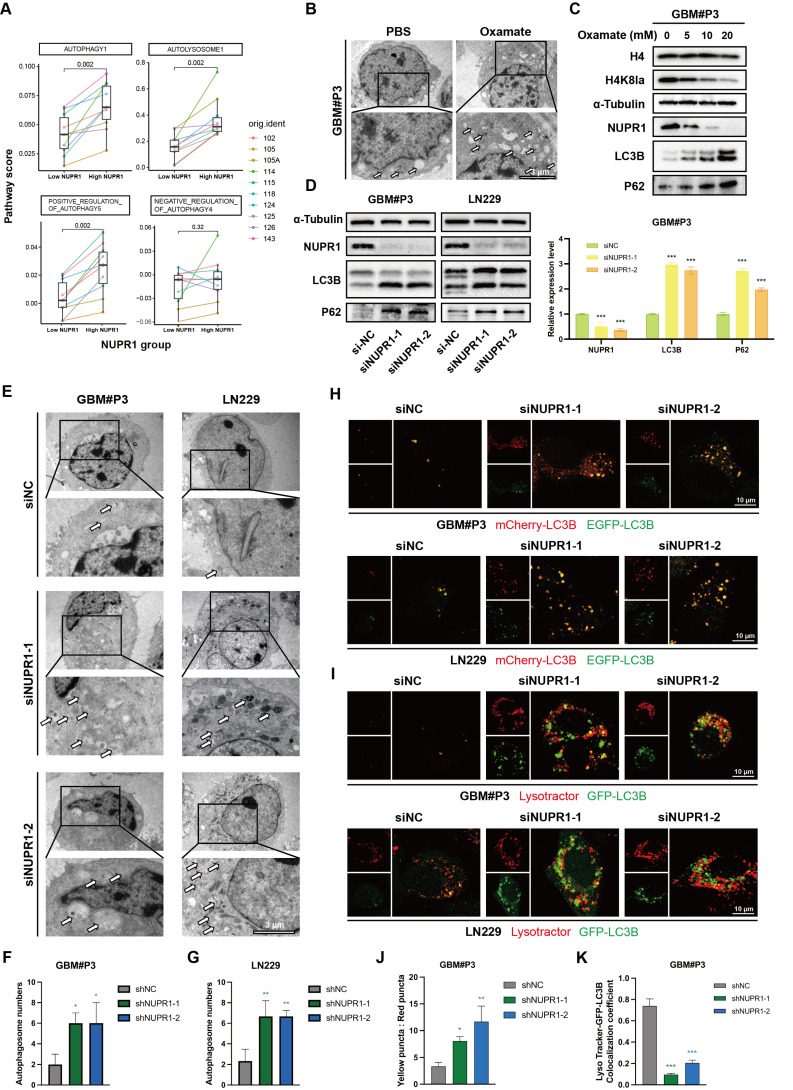
** NUPR1 knockdown induces autophagosome accumulation by impairing autolysosome formation.** (A) Differences in autophagy pathway scores between cells with high and low NUPR1 expression. (B) TEM images and statistical results showing the changes in the number of autophagosomes in GBM#P3 cells after oxamate treatment (scale bars: 3 μm). (C) Western blot analysis of LC3B and p62 expression in NUPR1-knockdown LN229 and GBM#P3 cells with quantification of grayscale values. (D) Western blot analysis of H4K8la, NUPR1, LC3B and p62 expression after treatment with different concentrations of oxamate. (E-G) TEM images and statistical results of the autophagosomes in LN229 and GBM#P3 cells after siNC and siNUPR1 treatment (scale bars: 3 μm). (H) Fluorescence images of LN229 and GBM#P3 cells transfected with the mCherry-EGFP-LC3B reporter. Cells were treated with siNC or siNUPR1 (scale bars: 10 μm). (I) Fluorescence images of GFP-LC3B and LysoTracker Red in LN229 and GBM#P3 cells treated with siNUPR1 or siNC (scale bars: 10 μm). (J) Statistical results of the ratio of autophagosomes to autolysosomes (yellow puncta/red puncta). (K) Analysis of the colocalization of lysosomes and autophagosomes in GBM#P3 cells. Data are presented as mean ± SD. Significance levels between specified treatment groups are indicated as follows: *P < 0.05, **P < 0.01, ***P < 0.001.

Prior research has established a strong link between NUPR1 and autophagy in tumor cells 41-43. Differential analysis of autophagy pathways based on single cell analysis suggested broad upregulation of autophagy and increased lysosomal activity in High-NUPR1 cells (Figure 6A). Meanwhile, cells are more inclined to undergo positive autophagic regulation, while other autophagy-related pathways also exhibit activation positively correlated with NUPR1 expression levels (Figure S6A). These findings imply a potential positive correlation between NUPR1 expression levels and the Warburg effect as well as autophagy activation in GBM cells. Furthermore, on the basis of the results of the KEGG enrichment analysis, we propose that H4K8kla-induced upregulation of NUPR1 expression likely influences malignant progression in GBM cells by modulating autophagy.

To further validate this hypothesis, changes in autophagosome numbers in GBM cells after oxamate treatment were examined and quantified using transmission electron microscopy (TEM) (Figure 6B and Figure S6B). The results indicated an increase in the number of autophagosomes following oxamate treatment, suggesting that reduced lactylation levels create conditions conducive to the accumulation of autophagosomes. We subsequently treated GBM#P3 cells with varying concentrations of oxamate and evaluated changes in the expression levels of NUPR1, as well as those of markers such as LC3B and p62, via Western blotting (Figure 6C). We subsequently performed Western blot analysis on GBM#P3 and LN229 cells treated with siNUPR1. The results revealed that NUPR1 knockout increased the protein levels of the autophagy marker LC3B, while SQSTM1/p62 (p62) levels were also elevated, suggesting impaired autophagic flux (Figure 6D). The results indicated that the expression of NUPR1 decreased with increasing concentrations of oxamate, while the corresponding accumulation of LC3B and p62 increased. Concurrently, TEM analysis revealed that NUPR1 knockdown induced autophagosome accumulation in both the LN229 and GBM#P3 cell lines (Figure 6E-G).

To further evaluate whether the alteration in NUPR1 expression affects the number of autophagosomes by increasing autophagic flux or blocking their fusion with lysosomes, we labelled the LC3B protein with an EGFP-mCherry probe. In this system, the acidic lysosomal environment quenches EGFP fluorescence while preserving the mCherry signal, allowing differentiation between autophagosomes (yellow puncta, mCherry+EGFP+) and autolysosomes (red puncta, mCherry+EGFP-). After knocking down NUPR1, we observed a significant increase in the number of yellow puncta (Figure 6H, J and Figure S6C). Next, we labelled lysosomes in LN229 and GBM#P3 cells with a LysoTracker lysosomal probe and labelled the LC3B protein in tumor cells with green fluorescent protein (GFP)-tagged adenovirus. Confocal microscopy revealed a significant reduction in the number of yellow puncta, which represent autolysosome formation (Figure 6I, K and Figure S6D), supporting the conclusion that a reduction in NUPR1 expression primarily increases LC3B levels and autophagosome counts by inhibiting autolysosome formation.

### NUPR1 knockdown impairs late-stage autophagy by blocking autophagosome-lysosome fusion

**Figure 7:**
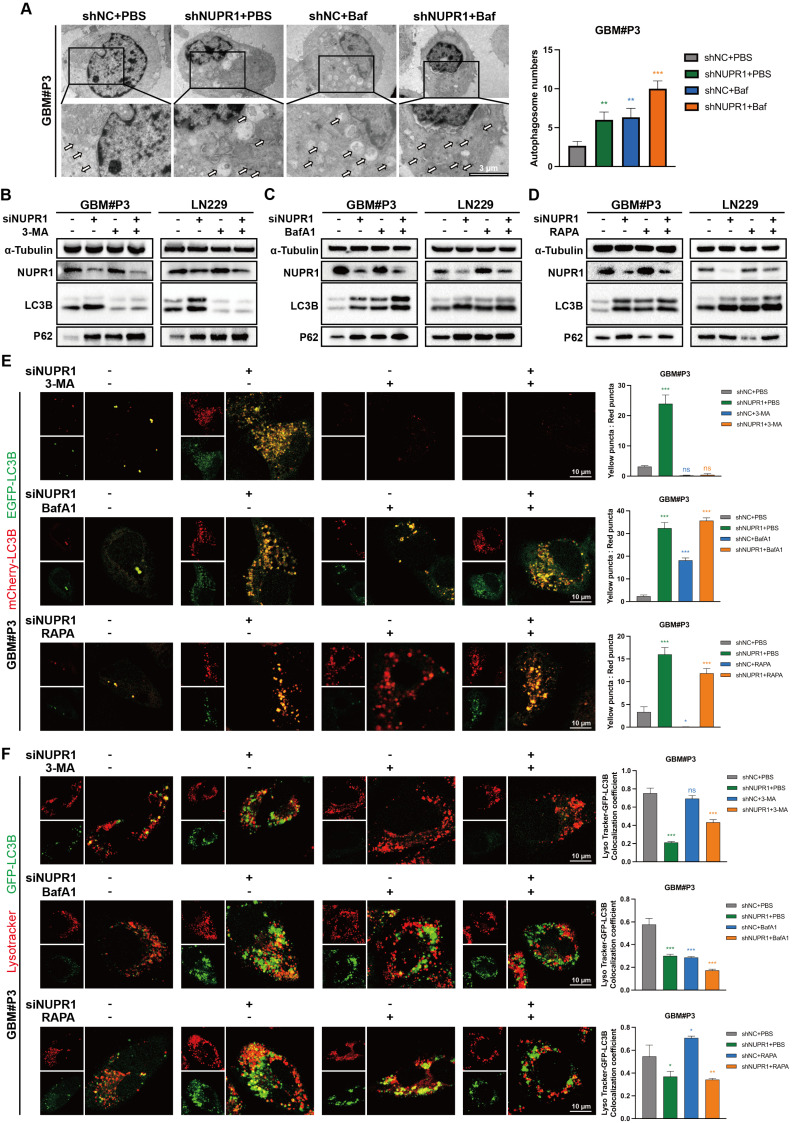
** NUPR1 deficiency blocks late-stage autophagy by inhibiting autophagosome‒lysosome fusion.** (A) Representative TEM images and quantitative analysis of autophagosome count in GBM#P3 cells under normal conditions and upon BafA1 treatment with NUPR1 knockdown (scale bars: 3 μm). (B-D) Western blot analysis of LC3B and p62 in NUPR1-knockdown cells treated with autophagy modulators (BafA1, 3-MA, Rapa). (E) Confocal microscopy analysis of fluorescence images of LN229 and GBM#P3 cells transfected with the mCherry-EGFP-LC3B reporter in cells treated with siNUPR1 and BafA1, 3-MA, or Rapa and the statistical results of the numbers of autophagosomes and autolysosomes (scale bars: 10 μm). (F) Representative fluorescence images and quantification of GFP-LC3B/LysoTracker Red colocalization in LN229 and GBM#P3 cells following treatment with siNUPR1, Baf, 3-MA, or Rapa (scale bars: 10 μm). Statistical results of the number of colocalized puncta of GFP-LC3B and LysoTracker. Data are presented as mean ± SD. Significance levels between specified treatment groups are indicated as follows: *P < 0.05, **P < 0.01, ***P < 0.001.

To investigate the functional relationship between NUPR1 and autophagic flux in GBM, transmission electron microscopy was employed to detect autophagosomes in GBM#P3 and LN229 cells following NUPR1 knockdown. Bafilomycin A1 (BafA1) treatment exacerbated the inhibition of autophagosome formation (Figure 7A and Figure S7A). The results indicated that in the presence of BafA1, the number of autophagosomes increased in response to the inhibition of late-stage autophagy. The effects of NUPR1 knockdown were similar, and the two factors had an additive effect, suggesting that impaired expression of NUPR1 may disrupt the normal progression of late-stage autophagy. To investigate the role of NUPR1 in autophagy regulation, we treated GBM#P3 and LN229 cells with different NUPR1 expression levels and pharmacological modulators of autophagy. Western blot analysis revealed that NUPR1 knockout increased the accumulation of LC3B-II and p62 induced by BafA1. Conversely, NUPR1 knockdown partially attenuated the reduction in LC3B expression caused by 3-methyladenine (3-MA), an early-stage autophagy inhibitor, while restoring p62 levels, which were decreased by rapamycin (Rapa) treatment (Figure 7B-D). Meanwhile, we also conducted a combined blocking validation targeting the key autophagy-regulating gene ATG7. Western Blot results showed that after knocking down ATG7 to block the normal formation of early autophagosomes, further knockdown of NUPR1 still led to additional accumulation of P62 to a certain extent, producing an effect similar to that of the 3-MA. This suggests that NUPR1 might primarily affect the late stages of autophagy (Figure S7B).

To further assess the impact of NUPR1 on autophagic flux, we performed mCherry/EGFP-LC3B reporter assays in treated cells. Our results demonstrated that NUPR1 deficiency specifically impaired the formation of autolysosomes without affecting autophagosome generation. The results indicated that after NUPR1 knockdown, the number of red puncta decreased and the number of yellow puncta increased in LN229 and GBM#P3 cells, similar to the effects observed with Baf treatment. Although 3-MA treatment initially reduced the number of autophagosomes, a slight recovery was observed after NUPR1 knockdown; however, these autophagosomes failed to fully integrate with the lysosomes. Additionally, this treatment partially inhibited the Rapa-induced increase in the number of autolysosomes (Figure 7E and Figure S7C). Next, we labelled lysosomes in LN229 and GBM#P3 cells using a LysoTracker lysosomal probe and marked the LC3B protein to analyse the formation of autophagosomes-lysosomes (Figure 7F and Figure S7D). The results indicated that both knockdown of NUPR1 and treatment with BafA1 resulted in similar outcomes, leading to the accumulation of autophagosomes and their impaired fusion with lysosomes. When autophagy initiation was blocked using 3-MA prior to NUPR1 knockdown, the numbers of both autophagosomes and autolysosomes significantly decreased. Conversely, when Rapa was applied to induce autophagy followed by NUPR1 knockdown, the increased number of autolysosomes was again reduced. These findings collectively indicate that NUPR1 knockdown disrupts late-stage autophagy by interfering with autophagosome‒lysosome fusion.

Finally, to determine whether NUPR1-driven autophagy is protective or lethal, LN229 and GBM#P3 cells were treated with varying NUPR1 expression levels combined with 3-MA and BafA1. CCK-8 assay's result indicated that autophagy inhibitors alone had a limited effect on tumor cell viability. However, when NUPR1 was combined with NUPR1 knockdown, glioma cell activity was significantly suppressed, suggesting that NUPR1-mediated activation of autophagic flux plays a protective role (Figure S7E-F).

Next, we investigated how NUPR1 regulates autophagosome-lysosome fusion. Based on existing research, NUPR1 has the ability to interact with other autophagy-related genes to regulate late-stage autophagy. Therefore, we first screened for its interacting proteins through immunoprecipitation and *in vitro* mass spectrometry (MS). Whole-cell lysates were immunoprecipitated using either anti-NUPR1 or control IgG antibodies. The eluted protein complexes were then analyzed by gel electrophoresis followed by Coomassie Blue staining and subsequent MS analysis. We focused on key late-stage autophagy regulatory genes (Table S4) and intersected them with the MS results, revealing a close interaction between VPS33A and NUPR1(Figure S8A). VPS33A is one of the four core subunits of the HOPS complex and is closely involved in the fusion process between autophagosomes and lysosomes 44. Studies have confirmed that the loss or mutation of VPS33A expression significantly impairs the formation of autolysosomes 45. Co-IP experiments confirm that NUPR1 exhibits strong binding affinity with VPS33A (Figure S8B). When NUPR1's expression is knockdown, we observed a corresponding downregulation in VPS33A levels (Figure S8C). This suggests that VPS33A may accumulate and interact with NUPR1 in response to its abnormal elevation, thereby promoting the degradation of autophagosomes.

### ZZW-115 inhibits glioma progression by targeting NUPR1-mediated autophagy

**Figure 8:**
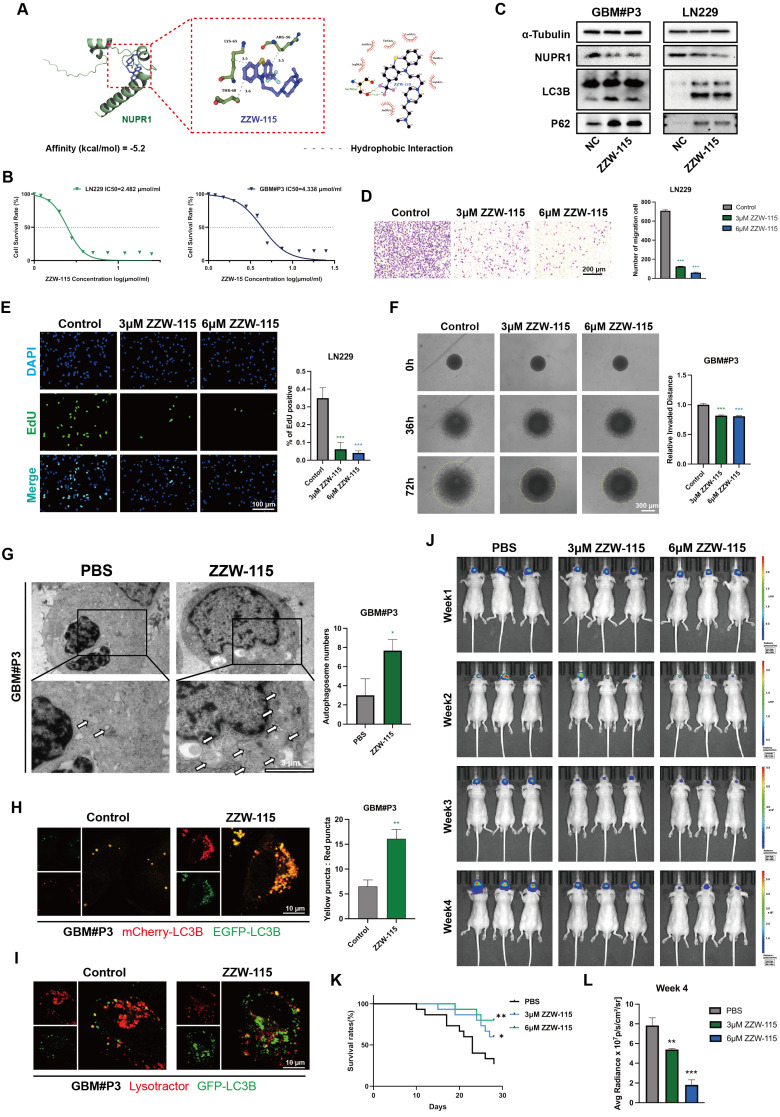
** The NUPR1 inhibitor ZZW-115 blocks autophagy and inhibits GBM progression *in vitro*.** (A) Molecular structure and molecular docking predictions of ZZW-115. (B) IC50 curves showing the response of GBM#P3 and LN229 cell lines to ZZW-115 treatment. (C) Western blot showing the NUPR1, LC3B and p62 levels in LN229 and GBM#P3 cells after treatment with ZZW-115. (D) Representative images and quantitative analysis of the Transwell invasion assays showing the invasive capacity of LN229 cells treated with 3 μM and 6 μM ZZW-115 (scale bars: 200 μm). (E) The effects of 3 μM and 6 μM ZZW-115 on the proliferation of LN229 cells were determined via an EdU staining assay with images and quantitative analysis (scale bars: 100 μm). (F) 3D spheroid invasion assay and statistical analysis of the inhibitory effect of ZZW-115 on long-distance invasion (scale bars: 300 μm). (G) TEM images showing autophagosome accumulation after ZZW-115 treatment and the statistical results of the number of autophagosomes (scale bars: 6 μm). (H) Fluorescence images of LN229 and GBM#P3 cells treated with ZZW-115 and PBS transfected with the mCherry-EGFP-LC3B reporter and analysis of the number of autophagosomes and autolysosomes (scale bars: 10 μm). (I) Fluorescence images and analysis of the colocalization of GFP-LC3B and LysoTracker Red in LN229 and GBM#P3 cells treated with ZZW-115 and PBS (scale bars: 10 μm). (J) Following intracranial implantation of luciferase-expressing GBM#P3 cells, mice were treated with PBS, 3 μM, or 6 μM ZZW-115. Tumor growth was monitored via IVIS imaging, and bioluminescence signals were measured at the indicated time points. (K). Overall survival was analyzed by Kaplan-Meier curves and compared using the log-rank test. (L). Bioluminescence values for assessing tumor growth on day 28. Data are presented as mean ± SD. Significance levels between specified treatment groups are indicated as follows: *P < 0.05, **P < 0.01, ***P < 0.001.

ZZW-115 is a small-molecule drug with significant NUPR1 inhibitory effects and can induce tumor cell death through necrosis and apoptosis. Through molecular docking predictions (Figure 8A), we further validated the binding ability of ZZW-115 with NUPR1 and preliminarily identified it as a NUPR1 inhibitor for subsequent therapeutic validation. We treated the GBM#P3 and LN229 cell lines with varying concentrations of ZZW-115 and plotted the IC50 curves (Figure 8B). The results indicated that the IC50 for the LN229 cell line was 4.438 μM/mL, while that for the GBM#P3 cell line was 2.482 μM/mL. On the basis of the IC50 results, we subsequently treated the two cell lines with high and low concentrations of ZZW-115, extracted the proteins, and performed Western blot experiments. ZZW-115 significantly suppressed NUPR1 expression and led to the accumulation of LC3B and p62 (Figure 8C).

We subsequently evaluated the *in vitro* cytotoxic effects of ZZW-115 on GBM through a series of cellular experiments. Transwell assays indicated that ZZW-115 treatment impaired the migration and invasive ability of GBM#P3 and LN229 cells (Figure 8D and Figure S8D-E). EdU staining confirmed that ZZW-115 treatment significantly reduced the proliferative capacity of GBM#P3 and LN229 cells, with higher concentrations of ZZW-115 exerting stronger inhibitory effects (Figure 8E). Additionally, the 3D tumor spheroid invasion assay demonstrated that different concentrations of ZZW-115 reduced the long-term invasiveness of GBM#P3 cells, with higher concentrations yielding more pronounced suppression (Figure 8F). Finally, a wound-healing assay verified that ZZW-115 treatment diminished the migratory and repair capabilities of LN229 tumor cells (Figure S8F). TEM results confirmed the accumulation of autophagosomes in ZZW-115-treated LN229 and GBM#P3 cells, which was consistent with the effects observed upon NUPR1 knockdown (Figure 8G and Figure S8H). To further verify whether ZZW-115 could inhibit autophagy by blocking the fusion of autophagosomes and lysosomes, we treated glioma cells labelled with mCherry/EGFP-LC3B probe with different concentrations of ZZW-115. ZZW-115 effectively reduced the fusion of autophagosomes and lysosomes (Figure 8H and Figure S8I-J). Additionally, validation through combined LysoTracker and GFP-LC3B adenovirus labelling confirmed that ZZW-115 inhibited the formation of autolysosomes in glioma cells (Figure 8I and Figure S8K).

### ZZW-115 inhibits glioma growth by suppressing the expression of NUPR1 in an orthotopic nude mouse model

To further evaluate the blood-brain barrier permeability of ZZW-115, we measured its brain concentration at different time points post-administration using LC-MS. The results indicated that ZZW-115 can achieve substantial accumulation in the brain within 1-2 h after administration (Figure S8G). We subsequently divided 45 nude mice into three groups. With the assistance of a stereotaxic instrument, we established orthotopic tumors using luciferase-stably labelled GBM#P3 cells. The mice then received intraperitoneal injections of ZZW-115 (n=30) or an equal volume of PBS (n=15). The nude mice in the ZZW-115 group were divided into two treatment groups, one group receiving high concentrations of ZZW-115 and the other receiving low concentrations of ZZW-115 (3 μM and 6 μM, respectively). Then we monitored tumor growth by bioluminescence imaging. The results indicated a significantly slower rate of tumor progression in the ZZW-115 treatment group compared to the PBS control group (Figure 8J). Moreover, high concentrations of ZZW-115 had the most pronounced tumor-suppressive effect in nude mice. K-M survival analysis showed a significant prognostic difference between the ZZW-115 and PBS treatment groups, with the high concentration of ZZW-115 again associated with superior prognostic outcomes (Figure 8K). Additionally, radiation intensity analysis indicated that the therapeutic efficacy of ZZW-115 was better than that of the PBS control (Figure 8L).

**Figure 9:**
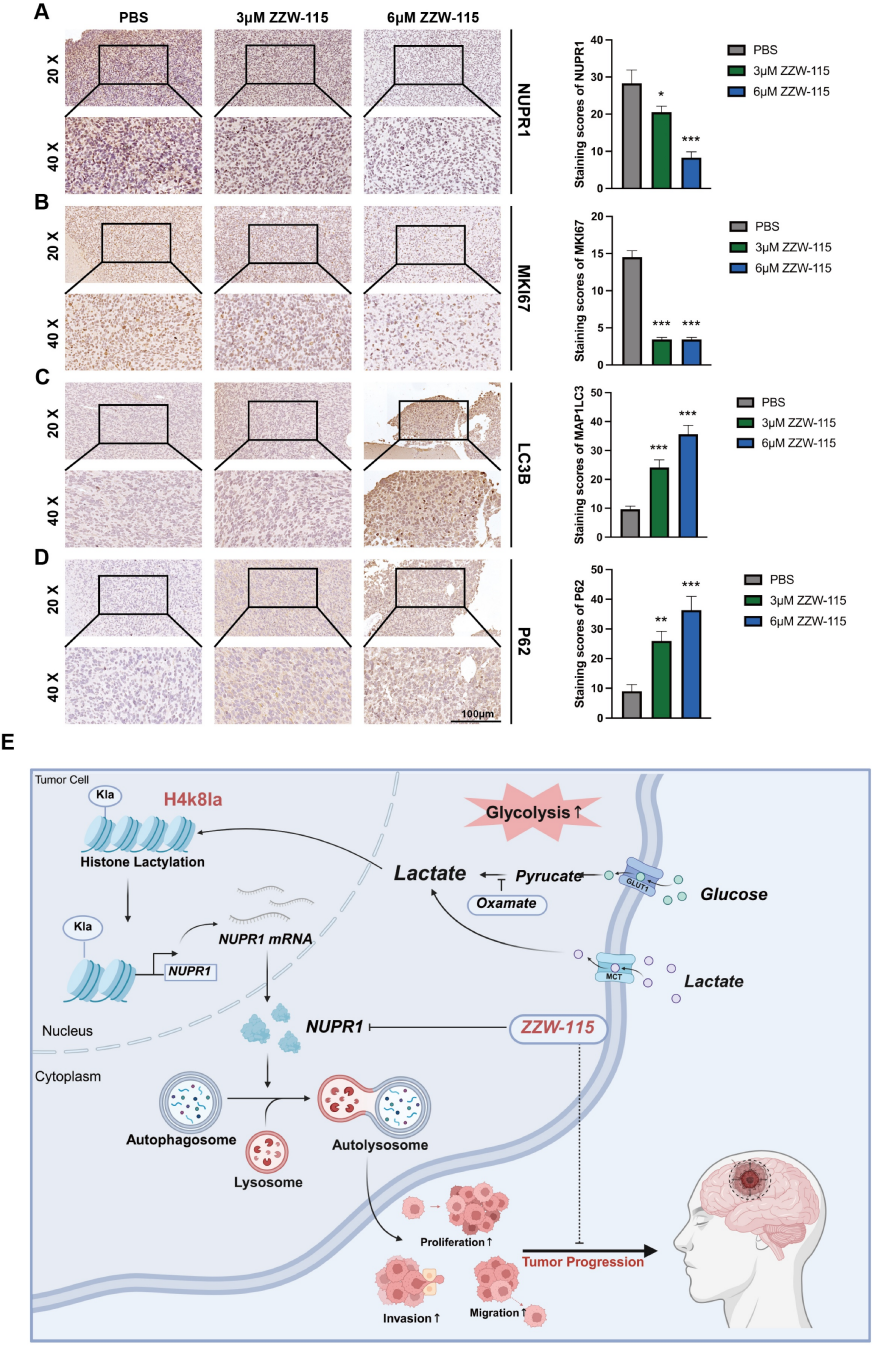
** ZZW-115 suppresses tumor growth and extends survival by targeting NUPR1 in an orthotopic GBM model.** (A-D) IHC staining images of MKi67, NUPR1, LC3B and p62 in tumor tissues across treatment groups, with statistical results of staining scores in the PBS, 3 μm ZZW-115 and 6 μm ZZW-115 groups (scale bars: 100 μm). (E) Study flowchart. Data are presented as mean ± SD. Significance levels between specified treatment groups are indicated as follows: *P < 0.05, **P < 0.01, ***P < 0.001.

The results of IHC staining for NUPR1 revealed that the ZZW-115 group exhibited low NUPR1 expression levels (Figure 9A), with the 6 μM group demonstrating the lowest expression. MKI67, a marker of proliferative capacity, was expressed at lower levels in the ZZW-115 group (Figure 9B). Furthermore, the expression level of MAP1LC3B was higher in the ZWW-115 than in the PBS group, with a greater increase observed in the higher concentration groups (Figure 9C). In contrast, this group had the highest expression level of p62 (Figure 9D). These results indicate that ZWW-115 significantly inhibits glioma cell proliferation and prolongs survival time *in vivo*. To summarize the experimental approach, a schematic workflow is provided in Figure 9E.

## Discussion

GBM remains among the most aggressive and lethal malignancies of the central nervous system 46. Metabolic dysregulation and profound cellular reprogramming are now recognized as hallmarks of GBM, contributing significantly to its rapid proliferation and invasive behaviour 47. These adaptations not only facilitate tumor survival under adverse conditions but also present promising therapeutic benefits. A cornerstone of GBM metabolic reprogramming is the Warburg effect, or aerobic glycolysis. This metabolic shift results in substantial lactate accumulation within the tumor microenvironment 48, 49. Consequently, targeting the Warburg effect has emerged as a promising strategy for GBM therapy 50.

Our research on GBM has revealed a significant accumulation of lactate, which is considered a substrate for lactylation modification, in the tumor microenvironment. Among these modifications, histone lactylation, which is closely associated with the Warburg effect and metabolic reprogramming, plays a particularly important role 51, 52. Several lactylation sites, such as H3K9la, H3K18la and H4K12la, play critical roles in the progression of various tumors and are significantly associated with poor clinical outcomes. For instance, H3K9la has been demonstrated to facilitate the progression of colorectal cancer through the induction of GRAMD1A expression 53. In GBM, the level of histone lactylation at the H3K9la site is also believed to be closely related to the development of temozolomide resistance and recurrence 54. This study aims to examine the dynamics of H4K8la lactylation in GBM. After oxamate was applied or LDH expression was inhibited to block lactate production and metabolism, the level of H4K8la in GBM cells was significantly decreased. Similarly, experiments using both *in vitro* and *in vivo* models demonstrated that when histone lactylation was suppressed, the proliferative, invasive, and migratory capacities of tumor cells were impaired to varying degrees. Exogenous lactate supplementation partially reversed this phenomenon. Additionally, the administration of oxamate improved the prognosis of model animals to some extent. These findings further support a clear negative correlation between H4K8la levels and GBM prognosis. Previous studies have suggested that histone lactylation modification sites with crucial biological functions typically involve closely associated modifying enzymes 55, 56. By conducting expression profiling and interaction analysis of known lactylation-modifying enzymes documented in the existing literature, we identified a significant association between the key epigenetic regulators P300 and HDAC2 and the H4K8la modification site. This finding not only indicates that lactylation modifications share common regulatory molecules with other modification types, such as acetylation, but also offers potential avenues for further investigation.

This study demonstrated that as the malignancy grade of glioma increases, lactate accumulation in tumor tissue becomes more pronounced and is accompanied by the upregulation of histone lactylation, particularly that of H4K8la. Such elevated lactylation levels are significantly correlated with poor clinical prognosis in GBM patients. In this study, enrichment analysis of CUT&Tag and transcriptome sequencing results following oxamate treatment revealed alterations in the autophagic process in GBM cells. As a highly promising therapeutic target for glioma, autophagy plays a critical role in the development of radioresistance and chemoresistance in GBM 57. Concurrently, blocking protective autophagy is considered a key strategy for inhibiting drug resistance in malignant tumors and modulating their metabolic reprogramming. Our previous experiments demonstrated that calnexin (CANX) can induce protective mitophagy, thereby promoting the emergence of TMZ chemoresistance in glioma cells 58. Previous research has confirmed that increased histone lactylation at promoter regions can induce the expression of target genes across various cell types. In the present study, an integrated analysis of Cut&Tag and transcriptome sequencing data after oxamate treatment revealed alterations in the autophagic process in GBM cells. By correlating these changes with lactylation-driven transcriptional regulation at promoter regions, we identified potential autophagy-related genes activated by H4K8la. Ultimately, NUPR1 was identified as a likely downstream target gene activated by this mechanism.

As a nuclear transcription regulator, NUPR1 orchestrates a variety of biological processes, including autophagy, the cell cycle, apoptosis, DNA repair, endoplasmic reticulum stress, the oxidative stress response, ferroptosis, and chromatin remodelling 59. NUPR1 has been implicated in tumor malignancy across cancer types, partly through its role in regulating autophagy. NUPR1 activates tumor-promoting autophagy mechanisms by binding to the promoters of autophagy-related genes, thereby mediating drug resistance and malignant progression in breast cancer 60. Giuseppa Augello *et al.* reported that Nupr1 knockdown leads to the accumulation of autophagy markers such as p62 and the inhibition of autophagic flux, thereby increasing the sensitivity of liver cancer to sorafenib 36. Zhang *et al.* reported that NUPR1 promotes the proliferation and invasion of oral squamous cell carcinoma (OSCC) by increasing the activity of the TFE3 (transcription factor E3) promoter and activating TFE3 transcription, thus maintaining autophagic flux. NUPR1 knockdown was shown to impair late-stage lysosomal function in OSCC cells, thereby inhibiting tumor progression 42. Studies in GBM suggest that NUPR1 inhibitors can increase tumor sensitivity to chemoradiotherapy 61.

With a focus on the regulatory role of NUPR1 in autophagy in GBM cells, we further elucidated its functional impact. TEM revealed that suppression of NUPR1 resulted in the pronounced accumulation of autophagosome-like structures. Consistent with these observations, Western blot analysis revealed the upregulation of both LC3B and SQSTM1/p62 expression upon NUPR1 inhibition. To discern whether these effects stemmed from enhanced autophagosome generation or impaired autophagic flux—specifically, a failure in autolysosomal degradation—we employed a pH-sensitive dual-fluorescent LC3 reporter system and performed live-cell imaging with LysoTracker-LC3B colocalization assays. Our results indicated that NUPR1 knockdown significantly disrupted the fusion of autophagosomes with lysosomes, thereby arresting autophagic flux. This blockage ultimately attenuated tumor cell proliferation and abrogated tumor cell invasive capacity. Further validation using a panel of autophagy modulators targeting distinct stages of the process confirmed that NUPR1 is critically involved in sustaining late-phase autophagy. Importantly, our data suggest that this autophagic activity has a protective effect on GBM cells, highlighting the context-dependent role of autophagy in glioma progression.

It has been demonstrated that NUPR1 knockdown suppresses the proliferation and metastatic potential of GBM cell lines. This effect primarily stemmed from the inhibition of autophagosome-lysosome fusion, a critical late-stage autophagic step, leading to disrupted autophagic flux and the consequent breakdown of protective autophagy. This phenomenon may be associated with the interaction between NUPR1 and VPS33A, as well as the functional execution of the HOPS complex regulated by VPS33A. Finally, we selected ZZW-115, a trifluoperazine derived compound known to inhibit NUPR1 62, as a potential therapeutic agent 63. Under normal conditions, NUPR1 functions to suppress PARP1 activity by physically interacting with it within the nucleus, as shown by molecular and cellular studies. Inactivation of NUPR1 triggers mitochondrial catastrophe, which profoundly affects cell survival. Previous studies in pancreatic cancer cells have demonstrated that treatment with ZZW-115 leads to complete disruption of mitochondrial function 35. The loss of mitochondrial membrane potential, coupled with a marked rise in superoxide production, ultimately mediated tumor cell death. These findings confirm that the expression level of NUPR1 is closely linked to the Warburg effect and lactylation-driven glycolytic metabolism in GBM cells. Moreover, ZZW-115 has been shown to further suppress OXPHOS in tumor cells, leading to a critical failure in energy supply 64. Thus, targeting GBM with ZZW-115 is supported by a solid theoretical rationale. ZZW-115 effectively suppressed GBM malignant progression in both *in vivo* and *in vitro* models. These findings indicate that NUPR1 maintains autophagic flux stability in GBM cells, facilitating tumor cell adaptation to microenvironmental stress, therapy resistance, and distant metastasis, highlighting its potential as a therapeutic target for GBM and further supporting the potential effectiveness of combining metabolic reprogramming and autophagy modulation as a therapeutic strategy against GBM.

This study has certain limitations. Although the results indicate that lactylation is involved in the malignant progression of GBM, the complex and dynamic nature of histone modifications indicates that single-base point mutations have limited impact. The H4K8la site focused on in this research is not the only site strongly associated with GBM development. Moreover, histone lactylation levels are regulated by numerous factors, which may be linked to key genetic mutations and subtypes of tumors, such as KRAS mutations in colorectal cancer 53. Research in this area remains relatively underexplored and should be a focus of subsequent studies. Although this study included a portion of glioma and normal brain tissue samples for clinical correlation analysis, gliomas are solid tumors with complex molecular subtypes. Factors such as IDH classification, 1p/19q co-deletion, and TERT promoter mutations all influence patient prognosis and treatment strategy selection. Additionally, some of the normal brain tissues in the study were sourced from craniotomy resections due to trauma or acute intracranial hypertension. Due to the effects of inflammatory infiltration and hypoxia-ischemia, the lactate accumulation levels in these brain tissues differ somewhat from those under physiological conditions. Further research will rely on larger-sample clinical studies to consolidate the reliability of the findings.

Additionally, the NUPR1-targeting inhibitor ZZW-115 investigated in this study can induce mitochondrial dysfunction and lead to metabolic collapse in tumor cells. This effect has demonstrated promising results in the killing of GBM cells both *in vitro* and *in vivo*. However, further clinical translation requires careful consideration of the drug's actual efficacy and potential side effects in patients with different glioma subtypes. Moreover, the optimal dosage and administration regimen should be determined through more detailed investigations. We also acknowledge the potential role of the microbiome and the gut-brain axis in tumor treatment evasion, which will be a focus of our future studies 65, 66. Overall, this study revealed a significant association between histone lactylation and protective autophagy, not only providing new insights into the pathogenesis of GBM but also opening up novel avenues for subsequent targeted therapy.

## Supplementary Material

Supplementary figures.

Supplementary table 1.

Supplementary table 2.

Supplementary table 3.

Supplementary table 4.

## Abbreviations

ATG: Autophagy-related
ANOVA: Analysis of Variance
BafA1: Bafilomycin A1
CCK-8: Cell Counting Kit-8
ChIP: Chromatin Immunoprecipitation
CNV: Copy Number Variation
CUT&Tag: Cleavage Under Targets and Tagmentation
DAPI: 4',6-Diamidino-2-Phenylindole
DMEM: Dulbecco's Modified Eagle Medium
EdU: 5-Ethynyl-2'-deoxyuridine
EGFP: Enhanced Green Fluorescent Protein
GBM: Glioblastoma
GEO: Gene Expression Omnibus
GFP: Green Fluorescent Protein
H4K8la: Histone H4 Lysine 8 lactylation
IHC: Immunohistochemistry
IF: Immunofluorescence
IC50: Half Maximal Inhibitory Concentration
KEGG: Kyoto Encyclopedia of Genes and Genomes
LDH: Lactate Dehydrogenase
LC3B: Microtubule Associated Protein 1 Light Chain 3 Beta
3-MA: 3-Methyladenine
NHA: Normal Human Astrocyte
NUPR1: Nuclear Protein 1 Transcriptional Regulator
NaLa: Sodium Lactate
OXPHOS: Oxidative Phosphorylation
PCA: Principal Component Analysis
PBS: Phosphate Buffered Saline
p62/SQSTM1: Sequestosome 1
RFP: Red Fluorescent Protein
siRNA: Small Interfering RNA
shRNA: Short Hairpin RNA
TEM: Transmission Electron Microscopy
UMAP: Uniform Manifold Approximation and Projection
