# Supplementary material for: Histone H4K8 lactylation promotes glioblastoma progression by inducing NUPR1-mediated autophagosome‒lysosome fusion
Source: Theranostics. 2026 Feb 4;16(8):4427–51. doi: 10.7150/thno.126579 (PMC12906151; doi:10.7150/thno.126579)
Supplement: Supplementary file 1 — Supplementary figures. [file thnov16p4427s1.pdf]

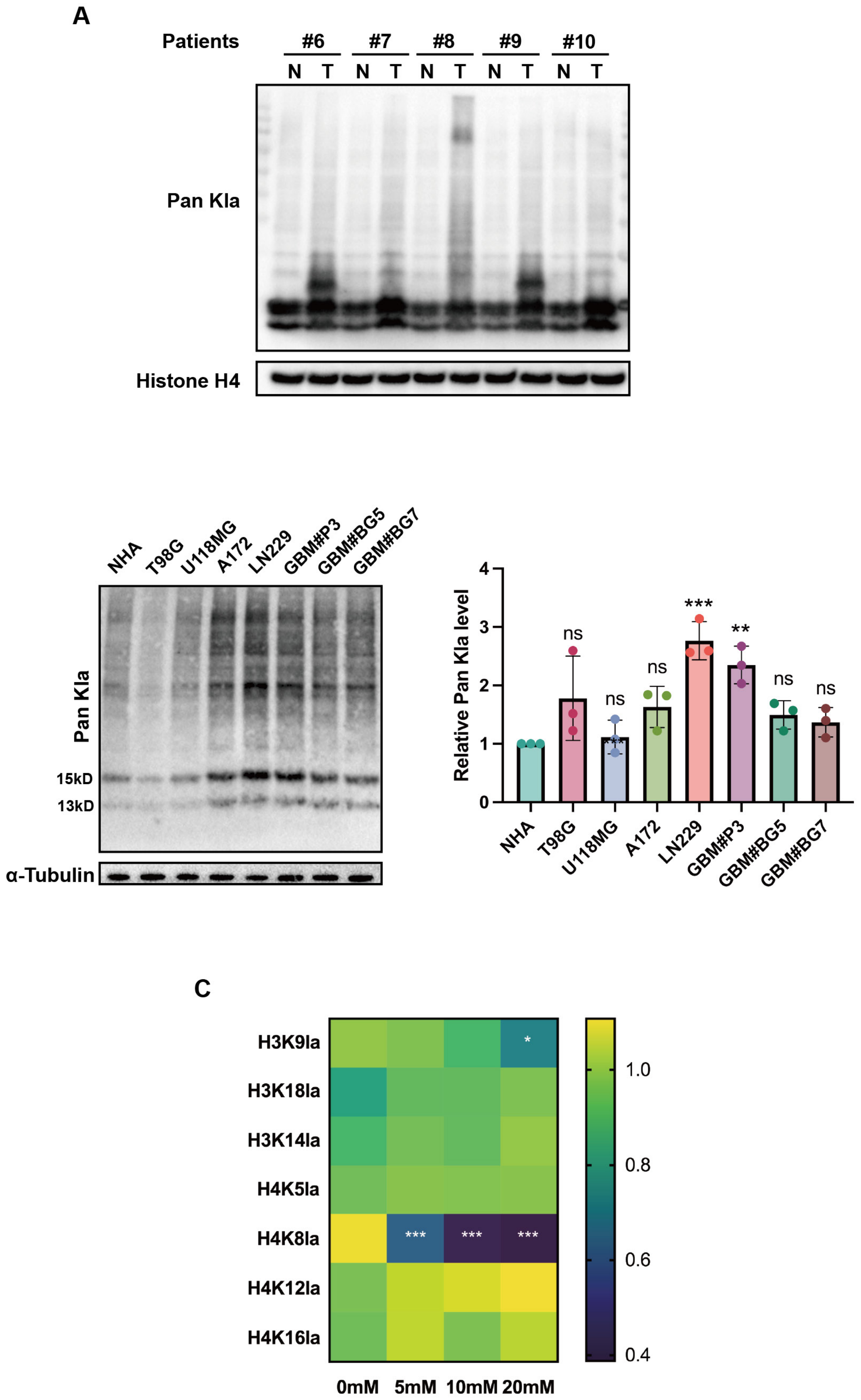

Figure S1. Identification of Pan-Lactylation and Histone Site Lactylation Levels

in GBM Cells (A) Pan-Kla levels in 5-paired GBM tissue and adjacent normal tissues were measured by Western blotting. (B) Western Blot shows the levels of pan-lactylation and quantitative analysis in different cell lines. (C) Heatmap displays the changes in lactylation levels at different histone sites in GBM#P3 cells as the concentration of oxamate varies. All data are expressed as the mean ± SD of values from experiments performed in triplicate. *P < 0.05, **P < 0.01, and ***P < 0.001 compared with the controls.

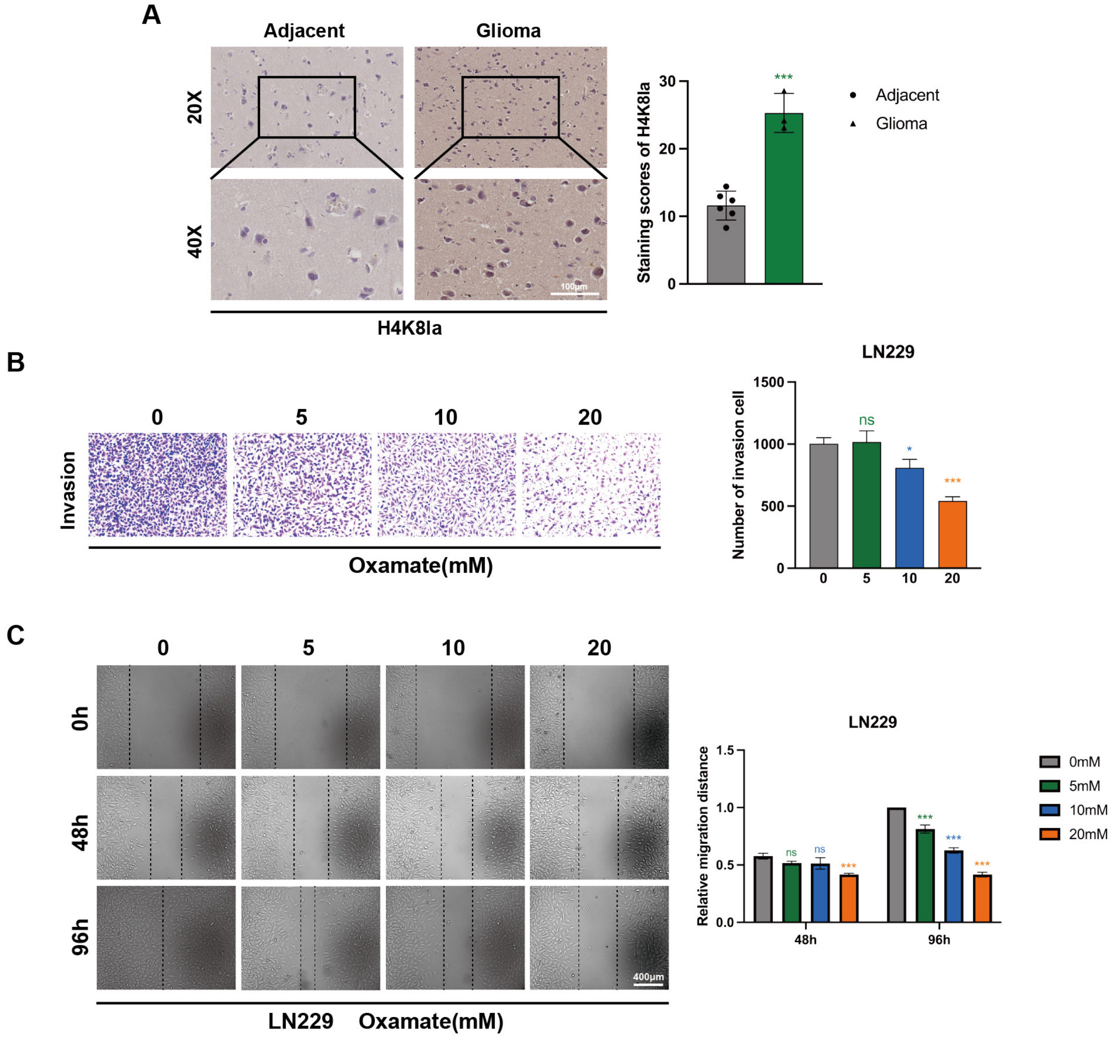

Figure S2.Elevated Lactylation Levels in GBM Suggest Active Malignant Tumor Progression (A) IHC demonstrates the difference in lactylation levels at the H4K8

site between peritumoral tissue and tumor tissue in GBM (scale bars: 100 μm). (B) Transwell assay reveals changes in the invasive ability of LN229 cells after oxamate treatment with a statistical graph (scale bars: 200 μm). (C) Scratch assay shows the healing status of LN229 cells after treatment with different concentrations of oxamate and statistical analysis at 48h and 96h in the scratch assay (scale bars: 400 μm). All data are expressed as the mean ± SD of values from experiments performed in triplicate. *$P < 0.05$, **$P < 0.01$, and ***$P < 0.001$ compared with the controls.

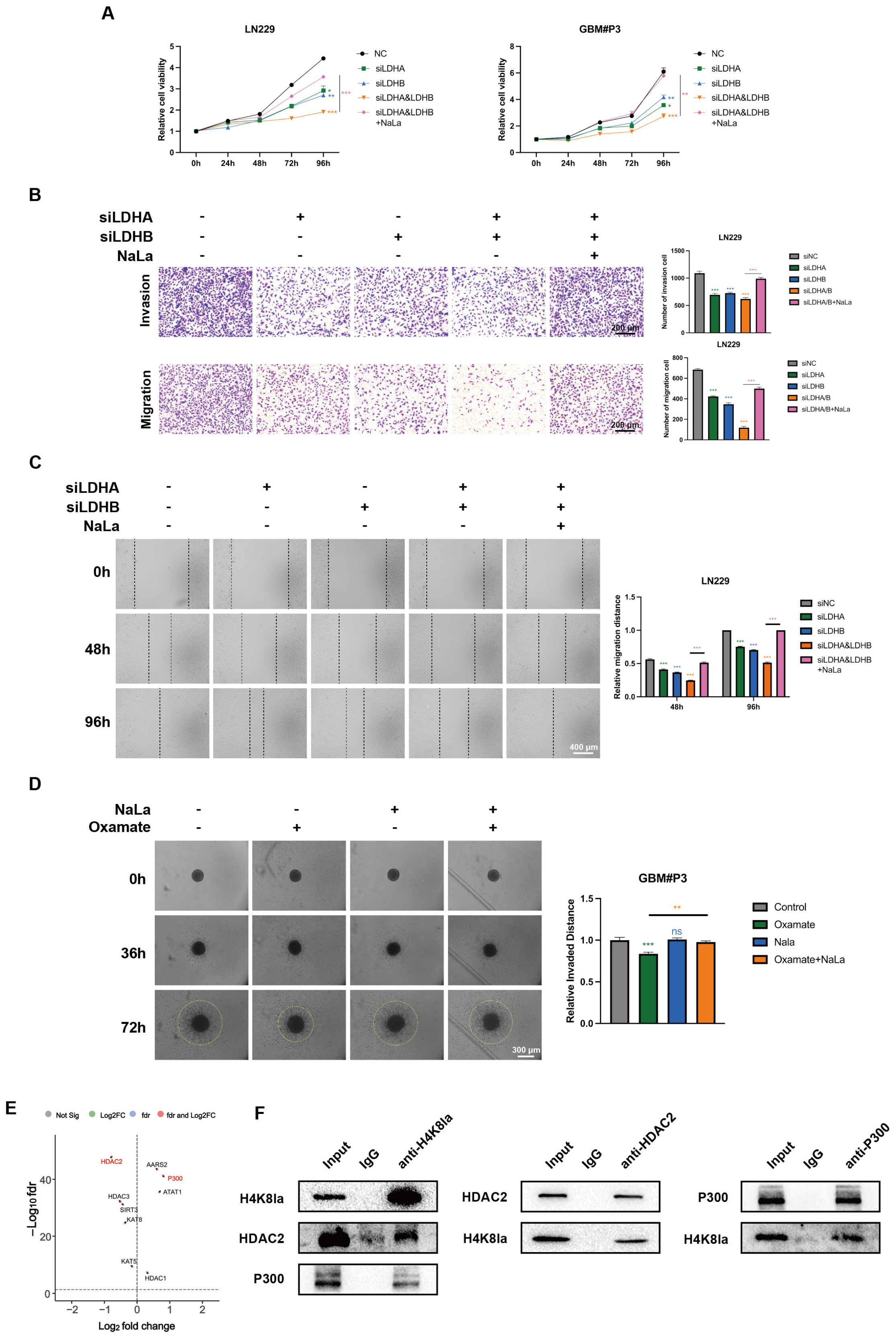

Figure S3. Impaired expression of key enzymes in lactate metabolism impairs the malignant progression of glioma cells. (A) CCK8 assays were used to analyse cell

viability after LDHA and/or LDHB knockdown and NaLa treatment. (B) Representative images and quantitative analysis of the Transwell invasion assays showing the migration and invasive capacity of LN229 cells after LDHA and/or LDHB knockdown and Nala treatment (scale bars: 200 μm). (C) Scratch assay shows the healing status of LN229 cells after treatment with LDHA and/or LDHB knockdown and NaLa (scale bars: 400 μm). (D) 3D invasive spheroid assay images and statistical analysis demonstrating the distant invasive capacity of GBM#P3 cells supplemented with NaLa following oxamate treatment (scale bars: 300 μm). (E) Volcano plot shows the expression differences of lactylation regulatory enzymes in gliomas compared to normal individuals. The data are shown as the means ± SDs and are representative of three independent experiments. (F) Co-immunoprecipitation (Co-IP) analysis confirms the physical interaction between H4K8la-P300 and H4K8la-HDAC2 in GBM#P3 cells. *P < 0.05; **P < 0.01, ***P < 0.001 between the two indicated treatments.

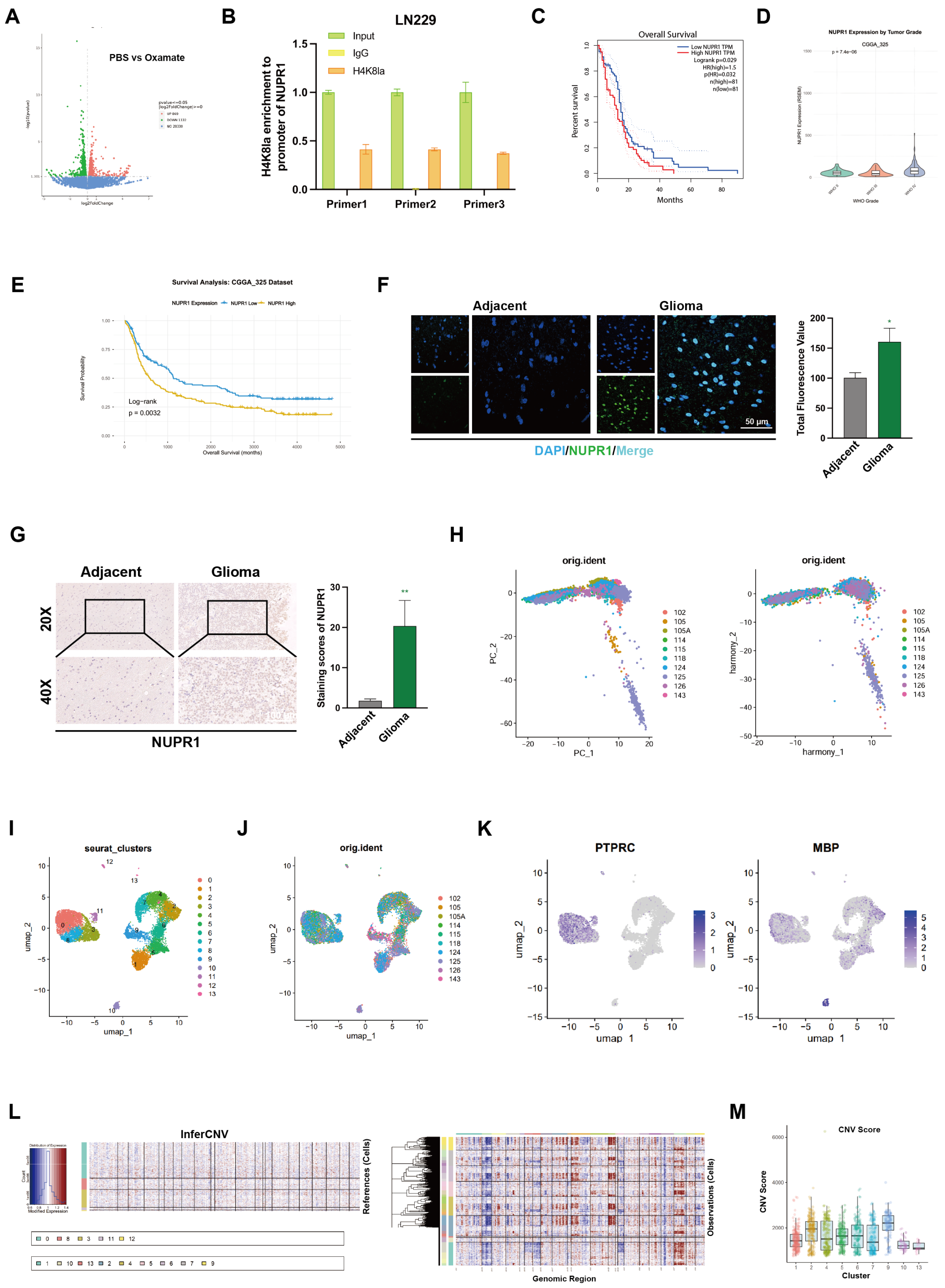

Figure S4 NUPR1 is an autophagy-related oncogene associated with H4K8la in glioma. (A)Volcano plot showing the differential gene expression between PBS and Oxamate treatment groups. (B) The statistical graph shows the qPCR results of DNA

fragments immunoprecipitated using H4K8la antibody after treating LN229 cells with oxamate. (C) Violin plots illustrate the differences in NUPR1 expression levels among glioma patients of different grades based on CGGA data. (D) KM curves based on CGGA data demonstrate the differences in survival periods among patients with varying NUPR1 expression levels. (E) KM curve illustrating the survival of glioma patients with different NUPR1 expression levels. (F) IF results displaying the expression and localization of NUPR1 in peritumoral and tumor tissues (scale bars: 50 μm). (G) IHC images and quantitative analysis showing differences in NUPR1 expression between peritumoral and tumor tissues (scale bars: 100 μm). (H) PCA distribution patterns of transcriptomic samples before (left) and after (right) batch correction. (I, J) UMAP plots showing the distribution of cells grouped by clusters and by samples. (K) Expression levels of PTPRC and MBP in glioblastoma tissues. (L) InferCNV showing copy number variations (CNVs) in all clusters. (M) CNV scores of cells in clusters 10 and 13 are lower than other clusters. All data are expressed as the mean ± SD of values from experiments performed in triplicate. $^{*}P < 0.05$, $^{**}P < 0.01$, and $^{***}P < 0.001$ compared with the controls.

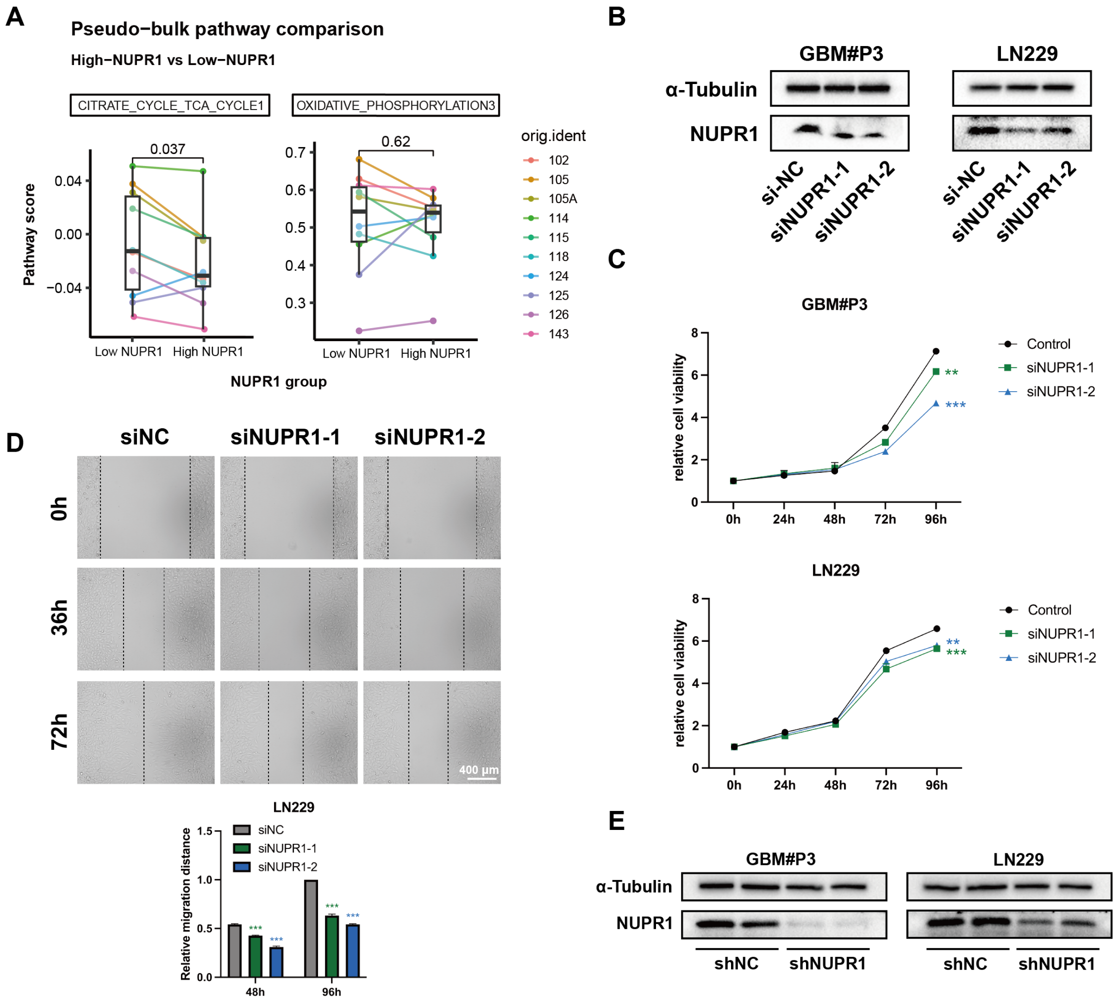

Figure S5. NUPR1 expression is regulated by lactylation and promotes GBM malignancy. (A) KEGG pathway activity scores demonstrate differences in metabolic pathways between glioma cells with high and low NUPR1 expression levels. (B) Analysis of the knockdown efficiency of two siRNAs targeting NUPR1 in LN229 and GBM#P3 cells via Western blotting. (C) A CCK8 assay was performed to analyse cell viability after the knockdown of NUPR1 in LN229 and GBM#P3 cells. (D) Representative images and quantitative analysis of wound-healing assays showing the tumour migration ability of LN229 cells treated with siNUPR1 (scale bars: 400 μm). (E) Western blot demonstrating the knockdown efficiency of shNUPR1. All data are expressed as the mean ± SD of values from experiments performed in triplicate.

*P < 0.05, **P < 0.01, and ***P < 0.001 compared with the controls.

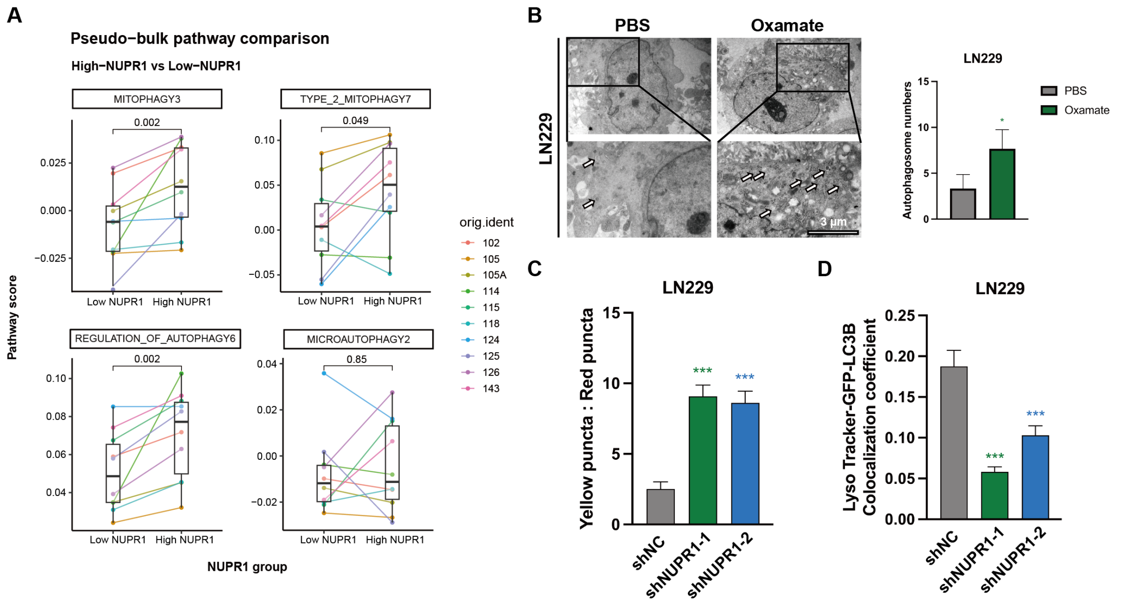

Figure S6 Changes in the autophagic activity of glioma cells after NUPR1 knockdown.

(A) Differences in autophagy pathway scores between cells with high and low NUPR1 expression. (B) TEM images and statistical results showing the changes in the number of autophagosomes in LN229 cells after oxamate treatment (scale bars: 3 μm). (C) The statistical results of the numbers of autophagosomes and autolysosomes in LN229 cells after siNUPR1 treatment. (D) Statistical analysis of the colocalization of GFP-LC3B and LysoTracker Red in LN229 treated with siNC and siNUPR1. The data are shown as the means ± SDs and are representative of three independent experiments. *P < 0.05; **P < 0.01, ***P < 0.001 between the two indicated treatments.

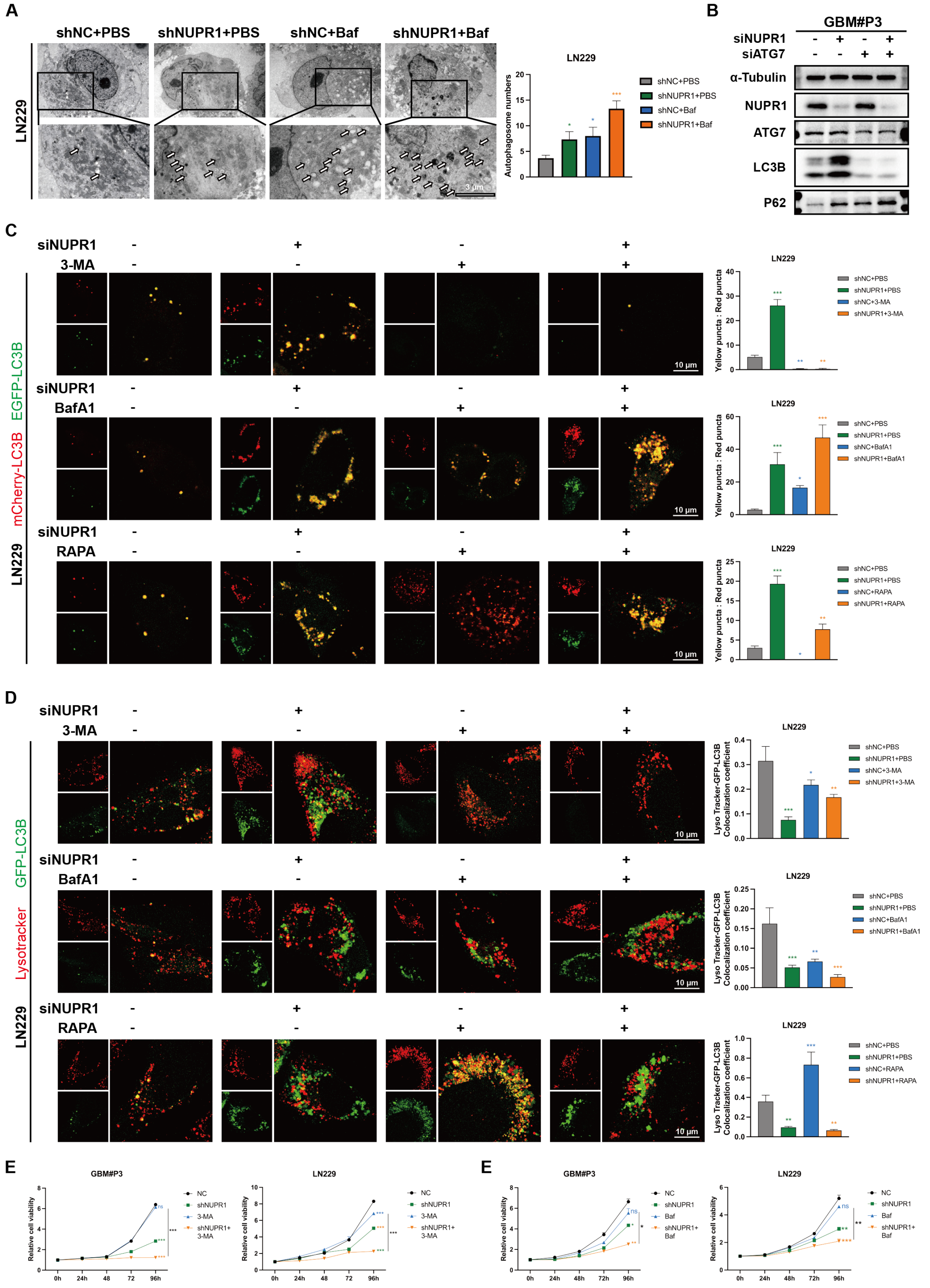

Figure S7 Knockdown of NUPR1 disrupts the protective autophagic flux within glioma cells. (A) Representative images and quantitative analysis of TEM images showing the changes in the number of autophagosomes in LN229 cells under normal

conditions and after Baf treatment with NUPR1 knockdown (scale bars: 3 μm). (B) Western blot analysis of LC3B and p62 in GBM#P3 cells treated with siATG7 and siNUPR1. (C) Confocal microscopy was used to analyze fluorescence images of LN229 cells transfected with the mCherry-EGFP-LC3B reporter following treatment with siNUPR1 in combination with Baf, 3-MA, or Rapa. Quantitative results showing the numbers of autophagosomes and autolysosomes are presented statistically (scale bars: 10 μm). (D) Fluorescence imaging and colocalization analysis of GFP-LC3B and LysoTracker Red in LN229 cells treated with siNUPR1 along with Baf, 3-MA, or Rapa. The number of GFP-LC3B and LysoTracker Red colocalized puncta was statistically quantified (scale bars: 10 μm). (E, F) The CCK-8 assay demonstrated the changes in viability of LN229 and GBM#P3 cells after NUPR1 knockdown under treatment with 3-MA and BafA1.The data are shown as the means ± SDs and are representative of three independent experiments. *$P < 0.05$; **$P < 0.01$, ***$P < 0.001$ between the two indicated treatments.

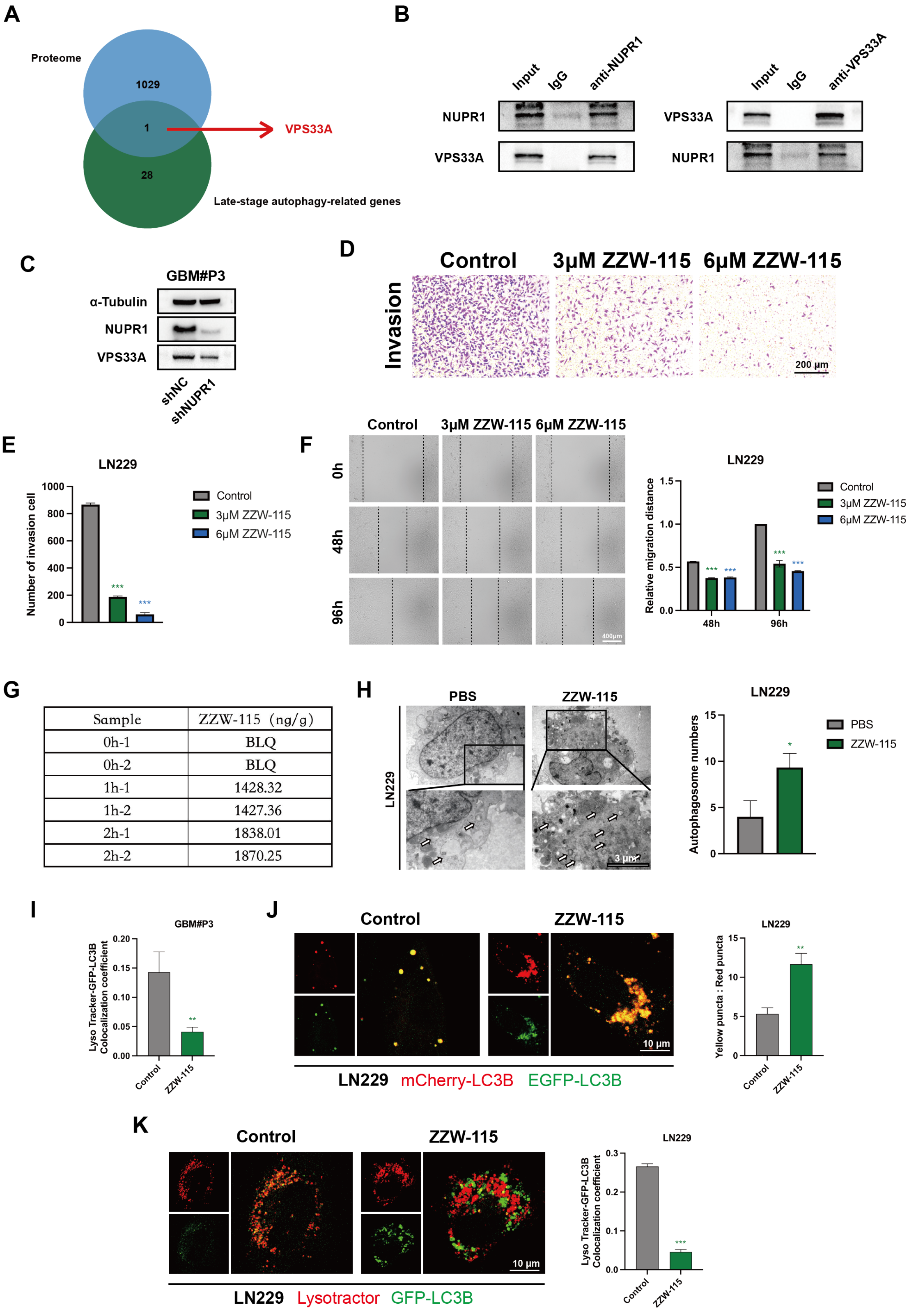

Figure S8.ZZW-115 inhibits the malignant progression of glioma by blocking

NUPR1-mediated autophagy (A) The Venn diagram displays the key late-stage autophagy genes co-precipitated in MS. (B) Co-IP analysis demonstrates the interaction between NUPR1 and VPS33A in GBM#P3 cells. (C) Western Blot shows the changes in protein expression of VPS33A after applying siNUPR1. (D, E) Transwell images and statistical graphs demonstrate the effects of different concentrations of ZZW-115 on the invasive ability of LN229 cells (scale bars: 200 μm). (F) Scratch assay and statistical analysis show changes in the migratory capacity of LN229 cells after treatment with different concentrations of ZZW-115 (scale bars: 400 μm). (G) The concentration of the drug in the brains of balb/c-nu mice after administration of 3 μM ZZW-115 was quantified using LC-MS spectrometry. Brain tissue samples were collected at three time points: 0, 1, and 2 hours. BLQ: indicates below the quantification limit. (H) TEM results and statistical analysis reveal alterations in autophagosomes in LN229 cells following ZZW-115 application (scale bars: 3 μm). (I) Statistical results of the number of colocalized puncta of GFP-LC3B and LysoTracker in GBM#P3 cells with PBS and ZZW-115 treatment. (J) Confocal microscopy analysis of fluorescence images of LN229 cells transfected with the mCherry-EGFP-LC3B reporter in cells treated with ZZW-115 and PBS and the statistical results of the numbers of autophagosomes and autolysosomes (scale bars: 10 μm). (K) Fluorescence images and analysis of the colocalization of GFP-LC3B and LysoTracker Red in LN229 cells treated with ZZW-115. Statistical results of the number of colocalized puncta of GFP-LC3B and LysoTracker (scale bars: 10 μm). The data are shown as the means ± SDs and are representative of three independent

experiments. *P < 0.05; **P < 0.01, ***P < 0.001 between the two indicated treatments.
